# Landscape of tumor suppressor long noncoding RNAs in breast cancer

**DOI:** 10.1186/s13046-019-1096-0

**Published:** 2019-02-14

**Authors:** Boran Pang, Qin Wang, Shipeng Ning, Junqiang Wu, Xingda Zhang, Yanbo Chen, Shouping Xu

**Affiliations:** 1 Department of Surgery, Rui Jin Hospital, Shanghai Key Laboratory of Gastric Neoplasm, Shanghai Institute of Digestive Surgery, Shanghai Jiao Tong University School of Medicine, Shanghai, 200025 China; 2 Department of Breast Surgery, Harbin Medical University Cancer Hospital, Harbin, 150040 China

**Keywords:** lncRNAs, Prognosis, Tumorigenesis, Breast cancer, EPB41L4A-AS2

## Abstract

**Background:**

The landscape and biological functions of tumor suppressor long noncoding RNAs in breast cancer are still unknown.

**Methods:**

Data from whole transcriptome sequencing of 33 breast specimens in the Harbin Medical University Cancer Center cohort and The Cancer Genome Atlas was applied to identify and validate the landscape of tumor suppressor long noncoding RNAs, which was further validated by The Cancer Genome Atlas pancancer data including 33 cancer types and 12,839 patients. Next, the expression model, prognostic roles, potential biological functions and epigenetic regulation of tumor suppressor long noncoding RNAs were investigated and validated in the breast cancer and pancancer cohorts. Finally, EPB41L4A-AS2 was selected to validate our novel finding, and the tumor suppressive roles of EPB41L4A-AS2 in breast cancer were examined.

**Results:**

We identified and validated the landscape of tumor suppressor long noncoding RNAs in breast cancer. The expression of the identified long noncoding RNAs was downregulated in cancer tissue samples compared with normal tissue samples, and these long noncoding RNAs correlated with a favorable prognosis in breast cancer patients and the patients in the pancancer cohort. Multiple carcinogenesis-associated biological functions were predicted to be regulated negatively by these long noncoding RNAs. Moreover, these long noncoding RNAs were transcriptionally regulated by epigenetic modification, including DNA methylation and histone methylation modification. Finally, EPB41L4A-AS2 inhibited breast cancer cell proliferation, migration and invasion and induced cell apoptosis in vitro*.* Mechanistically, EPB41L4A-AS2, acting at least in part as a tumor suppressor, upregulated tumor suppressor gene expression. Moreover, ZNF217 recruited EZH2 to the EPB41L4A-AS2 locus and suppressed the expression of EPB41L4A-AS2 by epigenetically increasing H3K27me3 enrichment.

**Conclusions:**

This work enlarges the functional landscape of known long noncoding RNAs in human cancer and provides novel insights into the suppressive roles of these long noncoding RNAs.

**Electronic supplementary material:**

The online version of this article (10.1186/s13046-019-1096-0) contains supplementary material, which is available to authorized users.

## Background

Breast cancer, which is the most common cause of death in women, has an incidence of 22.9% among all malignant tumors in women and causes 13.7% of cancer-related deaths among women with tumors [1–3]. Although different kinds of treatment, such as surgery, chemotherapy, hormonal therapy, radiotherapy and immunotherapy, have improved the prognosis of breast cancer patients to some extent, cancer-related death is still the most common cause of death [4, 5]. The main obstacles to breast cancer treatment arise from the insensitivity of cancer cells due to the overactivation of oncogenic signaling pathways, suppression of the expression of tumor suppressor genes or dormancy and subsequent reawakening of residual tumor cells, which may be a major problem with current treatment strategies [6–8]. Therefore, proper modulation of the expression of oncogenic or tumor suppressor genes is urgently needed to bypass the obstacles that appear in traditional breast cancer treatment.

As 75% of the genome is transcribed into noncoding RNAs, whether noncoding RNAs can serve as regulators in various biological processes has attracted increasing attention [9, 10]. Among the noncoding RNAs, long noncoding RNAs (lncRNAs) are defined as transcripts with little or no protein-coding capacity that are longer than 200 nucleotides [10, 11]. In a recent report, Li and his colleagues summarized the functional classification and experimental dissection of lncRNAs. LncRNAs participate in multiple biological processes, including histone methylation, gene transcription, protein translation, and posttranslational modification [9]. Moreover, lncRNAs have been indicated to be associated with diverse diseases, such as Alzheimer’s disease, infectious diseases, and cancer [11]. However, the comprehensive understanding of lncRNAs in cancer is still incomplete, and the exploration of the specific roles of lncRNAs in cancer is urgently needed.

In our previous study, the oncogenic lncRNA landscape was identified and validated in breast cancer [10]. Moreover, we also identified an lncRNA, linc00152, which functions as a tumor propellant in breast cancer and pan-cancer [11]. In addition, lncRNAs acting as oncogenic drivers have been reported in breast cancer and other cancers [12–15]. Lnc-BM functions as a JAK2-binding lncRNA and promotes breast cancer brain metastasis [13]. LNMAT1 has been proven to promote lymphatic metastasis via CCL2-dependent macrophage recruitment [14]. LncGata6 can maintain cell stemness and promote tumorigenesis [15]. In contrast, few lncRNAs that function as tumor suppressors have been reported in breast cancer. Of these reported lncRNAs, EPB41L4A-AS2 and EGOT were first reported to suppress proliferation in breast cancer cells [16, 17]. Other lncRNAs, including GAS5 [18, 19], ZFAS1 [20], XIST [21, 22], and MEG3 [23–25], function as tumor suppressors. However, there is no comprehensive report on the landscape of tumor suppressor long noncoding RNAs (TSLNRs) in breast cancer.

In this study, we first defined the lncRNAs that are downregulated in cancer tissue, are correlated with favorable prognosis and function as tumor suppressors in breast cancer as TSLNRs. We identified and validated the comprehensive landscape of TSLNRs in breast cancer via whole transcriptome sequencing of the Harbin Medical University Cancer Center (HMUCC) cohort and The Cancer Genome Atlas (TCGA) breast cancer and pancancer data. The expression of the TSLNRs was downregulated in cancer tissue compared with normal tissue, and the expression of some TSLNRs was negatively associated with TNM stage in the pancancer data. Next, the TSLNRs were found to be correlated with a favorable prognosis in breast cancer patients and the patients in the pancancer cohort. Moreover, the TSLNRs were regulated by epigenetic modification, including DNA methylation and histone methylation modification. Finally, EPB41L4A-AS2, one of the validated TSLNRs, was selected to validate our novel finding, and its tumor suppressor roles in breast cancer were examined. As expected, EPB41L4A-AS2 inhibited breast cancer cell proliferation, migration and invasion and induced cell apoptosis partly via the upregulation of RARRES1 expression. In addition to mediating the DNA methylation regulation of EPB41L4A-AS2, ZNF217 recruited EZH2 to the EPB41L4A-AS2 locus and suppressed the expression of EPB41L4A-AS2 by epigenetically increasing H3K27me3 enrichment.

## Materials and methods

### Clinical sample collection and whole transcriptome sequencing

At the HMUCC, 33 samples including 15 breast cancer tissue samples, 15 adjacent normal tissue samples and 3 normal breast tissue samples were obtained from patients who had not received chemotherapy or radiotherapy. The protocol conformed to clinical research guidelines and was approved by the research ethics committee of HMUCC. Written informed consent was obtained from all of the patients who participated in this study. For whole transcriptome sequencing, ribosomal RNA was removed using Ribo-Zero™ Gold kits (Epicentre, Wisconsin, USA). Sequencing libraries were generated according to the manufacturer’s recommendations with varied index labeling using the NEBNext® Ultra™ Directional RNA Library Prep Kit for Illumina (New England Biolabs, Ipswich, USA). The libraries were then sequenced on an Illumina HiSeq 2500 platform, and 100-bp paired-end reads were generated. The raw sequencing data from this study have been deposited in the NCBI Gene Expression Omnibus (GEO) database under accession number GSE71651 (https://www.ncbi.nlm.nih.gov/geo/query/acc.cgi?acc = GSE71651s).

### Access and analysis of public data

The breast cancer and pancancer genome-wide gene profiles were downloaded from TCGA (https://tcga-data.nci.nih.gov/). All transcripts were normalized by the log_2_ method. The correlations between genes were assessed by Spearman’s correlation coefficients according to the protocol described in a previous study [26]. Unpaired Student’s t-tests were used to detect significant differences among tumors or between tumor and normal samples. Guilt-by-association analysis was performed to identify genes that were positively and negatively correlated with lncRNA expression [9, 27, 28]. During this analysis, the TCGA data were evaluated to generate a pairwise Spearman’s correlation between the expression of an lncRNA and that of all coding genes [10]. Only associated coding genes with an absolute r ≥ 0.4 and a statistically significant correlation (*P* < 0.05) were retained. Gene ontology (GO) term enrichment and Kyoto Encyclopedia of Genes and Genomes (KEGG) pathway analysis of these genes were performed using DAVID as previously described [29, 30]. H3K27me3 chromatin immunoprecipitation (ChIP)-seq data from Encyclopedia of DNA Elements (ENCODE) (www.encodeproject.org/) were analyzed. BATMAN-TCM (http://bionet.ncpsb.org/batman-tcm/) and PharmMapper (http://59.78.96.61/pharmmapper) were applied for pharmacological network analysis [31–33]. DNA methylation data were downloaded from the Illumina Infinium HumanMethylation450 Beadchip dataset in TCGA (https://tcga-data.nci.nih.gov/). The DNA methylation level ranged from 0 (least methylated) to 1 (most methylated). The methylation level was calculated as beta = Methylated probe intensity (M)/(Unmethylated probe intensity (U) + Methylated probe intensity (M) + 100) according to the method described in our previous study [11].

### Cell culture

UACC812, BT549 and MDA-MB-453 cells were cultured in RPMI-1640 medium or DMEM (Gibco, Carlsbad, CA, USA). All media were supplemented with 10% fetal bovine serum (FBS). All cells were incubated at 37 °C in humidified air containing 5% CO_2_. Thereafter, all cells were cultured in regular medium for 24 h before receiving the appropriate treatment.

### Plasmid and lentivirus production and infection

For the knockdown of EPB41L4A-AS2 expression, two human EPB41L4A-AS2-targeted RNAi sequences (RNAi #1225 sense: TTGATGGAGTTTCCTCTCATA; RNAi #1225 antisense: TATGAGAGGAAACTCCATCAA; RNAi #1237 sense: TTGATGGAGTTTCCTCTCATA; and RNAi# 1237 antisense: TATGAGAGGAAACTCCATCAA) were obtained from GeneChem, Co., Ltd. (Shanghai, China). A scrambled negative control siRNA and target gene siRNAs were purchased from Invitrogen (Invitrogen, CA, USA). The siRNAs were transfected into cells using Lipofectamine 3000 (Invitrogen, CA, USA) according to the manufacturer’s protocol. In addition, lentiviral particles were constructed and packaged by Shanghai GeneChem, Co., Ltd. Briefly, cells were infected with lentivirus to generate stable cell lines. After 24 h, the cells were transferred to medium containing 4 μg/ml puromycin and cultured for 3 days.

### Cell viability assays

Cell viability assays were performed using the Cell Counting Kit-8 (CCK-8; Dojindo Laboratories, Kumamoto, Japan) assay according to the manufacturer’s instructions and as previously described [21]. Briefly, cells were plated at a density of 5 × 10^3^ cells/well in 96-well plates. CCK-8 solution was added to each well and incubated at 37 °C for 90 min. Then, the absorbance of the cell suspension was measured at a wavelength of 450 nm. Medium containing 10% CCK-8 served as a negative control.

### Colony formation assays

Cells were plated into a 6-well plate and cultured in medium containing 10% FBS for two weeks. Colonies were fixed with methanol for 30 min, and 500 μl of 0.5% crystal violet was subsequently added (Sigma-Aldrich, St. Louis, MO, USA) to each well for 30 min for visualization and counting.

### Wound healing assays and invasion assays

For wound healing assays, cells were seeded in plates in DMEM containing 1% FBS. Monolayers were wounded with a sterile 10-μl pipette tip, and phosphate-buffered saline (PBS) was applied to remove the suspended cells. Images were captured of each well at different time points. The migration distance was estimated based on the width of the wounds using ImageJ software. For invasion assays, cells were seeded in plates coated with Matrigel (Sigma-Aldrich, USA) according to the manufacturer’s instructions. Medium containing 10% FBS was added to the lower chamber for 24 h. Cotton swabs were used to remove the noninvading cells that remained in the top chamber, and the cells that had invaded to the underside of the membrane were fixed in 100% methanol for 30 min and stained with 0.5% crystal violet. Finally, images were obtained under a light microscope.

### RNA preparation and qRT-PCR

Total RNA was extracted using TRIzol Reagent according to the manufacturer’s protocol (Invitrogen, Beijing, China), and cDNA was synthesized using the PrimeScript RT Reagent Kit with gDNA Eraser (Takara Bio, Otsu, Japan). mRNA expression was examined by real-time PCR using FastStart Universal SYBR Green Master Mix (Roche, Mannheim, Germany) with gene-specific primers and the ABI 7500 Fast Real-time PCR Detection System (Applied Biosystems, Foster City, CA, USA). The results were normalized to the expression of GAPDH. The primer sequences were as follows: EPB41L4A-AS2-F: 5′-CGGAGCAGGTGCAATCTGT-3′; EPB41L4A-AS2-R: 5′-CCCTCGTGTCTCCCCTAACTG-3′; RARRES1-F: 5′-AAACCCCTTGGAAATAGTCAGC-3′; RARRES1-R: 5′-GGAAAGCCAAATCCCAGATGAG-3′; ST18-F: 5′-CAAACCACCTAGAGTCCCAAAG-3′; ST18-R: 5′-ACACCTGTTCTCACAAGGGATA-3′; BMP4-F: 5′-CAAACCACCTAGAGTCCCAAAG-3′; BMP4-R: 5′-GACGGCACTCTTGCTAGGC-3′; FOXA2-F: 5′-GGAGCAGCTACTATGCAGAGC-3′; FOXA2-R: 5′-CGTGTTCATGCCGTTCATCC-3′; FOXL1-F: 5′-GCCTCGCCCATGCTGTATC-3′; FOXL1-R: 5′-CGTTGAGCGTGACCCTCTG-3′; LRIG1-F: 5′- GGACTTGCCGAACCTACAGG-3′; LRIG1-R: 5′-GCTGCGAATCTTGTTGTGCTG-3′; RASSF1-F: 5′-ATGTGCCTACCTGATACTTT-3′; RASSF1-R: 5′-ATGGTGAACCTGGAGAAC-3′; GAPDH-F: 5′-CATGTTCGTCATGGGTGTGAA-3′; and GAPDH-R: 5′-GGCATGGACTGTGGTCATGAG-3′.

### Flow cytometry analysis

Flow cytometry analysis was performed according to the manufacturer’s protocol. Cells were seeded in 6-well plates for 24 h, harvested and washed twice with cold PBS. Then, the cells were stained with PE-conjugated Annexin V and 7-AAD (BD Biosciences, San Jose, CA, USA) for 15 min at room temperature in the dark. Finally, the cells were analyzed using a FACSCalibur flow cytometer (BD Biosciences, San Jose, CA, USA).

### Co-IP and western blot assays

Coimmunoprecipitation (Co-IP) and western blot assays were carried out by using the Pierce™ Crosslink Magnetic IP/Co-IP Kit (Thermo Fisher Scientific, USA) according to the manufacturer’s protocol [34]. Anti-ZNF217 (ab117798) and anti-EZH2 (ab186006) antibodies were obtained from Abcam. An anti-tubulin (sc-73,242, 1:1000) antibody was purchased from Santa Cruz Biotechnology. First, cells were lysed with lysis buffer and 2% SDS. Next, the protein concentrations were evaluated using a protein assay kit (Bio-Rad, Richmond, CA), and equal amounts of protein were separated by SDS-PAGE, followed by electroblotting onto a nitrocellulose membrane, which was then blocked with 5% nonfat milk in 0.1% Tween 20-PBS overnight at 4 °C. The membrane was immunoblotted with primary antibodies against Bcl-2 (ab32124), Bax (ab182733) and RARRES1 (ab76530). After washing with Tween 20/PBS, the membrane was incubated with horseradish peroxidase-conjugated secondary antibodies for 1 h. Finally, protein bands on the membrane were visualized by an enhanced chemiluminescence western blotting detection system (Western Lightning; Perkin-Elmer, Norwalk, CT).

### Chromatin immunoprecipitation assays

Chromatin immunoprecipitation (ChIP) assays were performed using the ChIP Assay Kit (Beyotime, Shanghai, China) according to the manufacturer’s protocol with slight modifications. Cells were crosslinked with 1% formaldehyde, and this reaction was terminated after 10 min by the addition of glycine at a final concentration of 0.125 M. DNA was immunoprecipitated from the sonicated cell lysates using anti-ZNF217 and anti-H3K27me3 antibodies(Abcam); IgG (BD Biosciences, San Diego, CA, USA) served as the negative control. The DNA was subjected to PCR to amplify the ZNF217 and H3K27me3 binding sites. The amplified fragments were then analyzed on an agarose gel. Chromatin (10%) prior to immunoprecipitation was used as the input control.

### Statistical analyses

Overall survival (OS) and disease-free survival (DFS) were calculated as the time from surgery until the occurrence of death and relapse, respectively. The expression of lncRNAs was dichotomized using the median expression as the cut-off to define high values (at or above the median) versus low values (below the median). The differences between groups in the results of the in vitro experiments were analyzed using Student’s t-test. Spearman’s correlation coefficients were calculated for correlation analyses. All of the experiments were performed independently in triplicate. R software was used to prepare all figures. All statistical tests were two-sided, and *P* < 0.05 indicated statistical significance.

## Results

### Discovery and validation of the TSLNR landscape in breast cancer

**Fig. 1:**
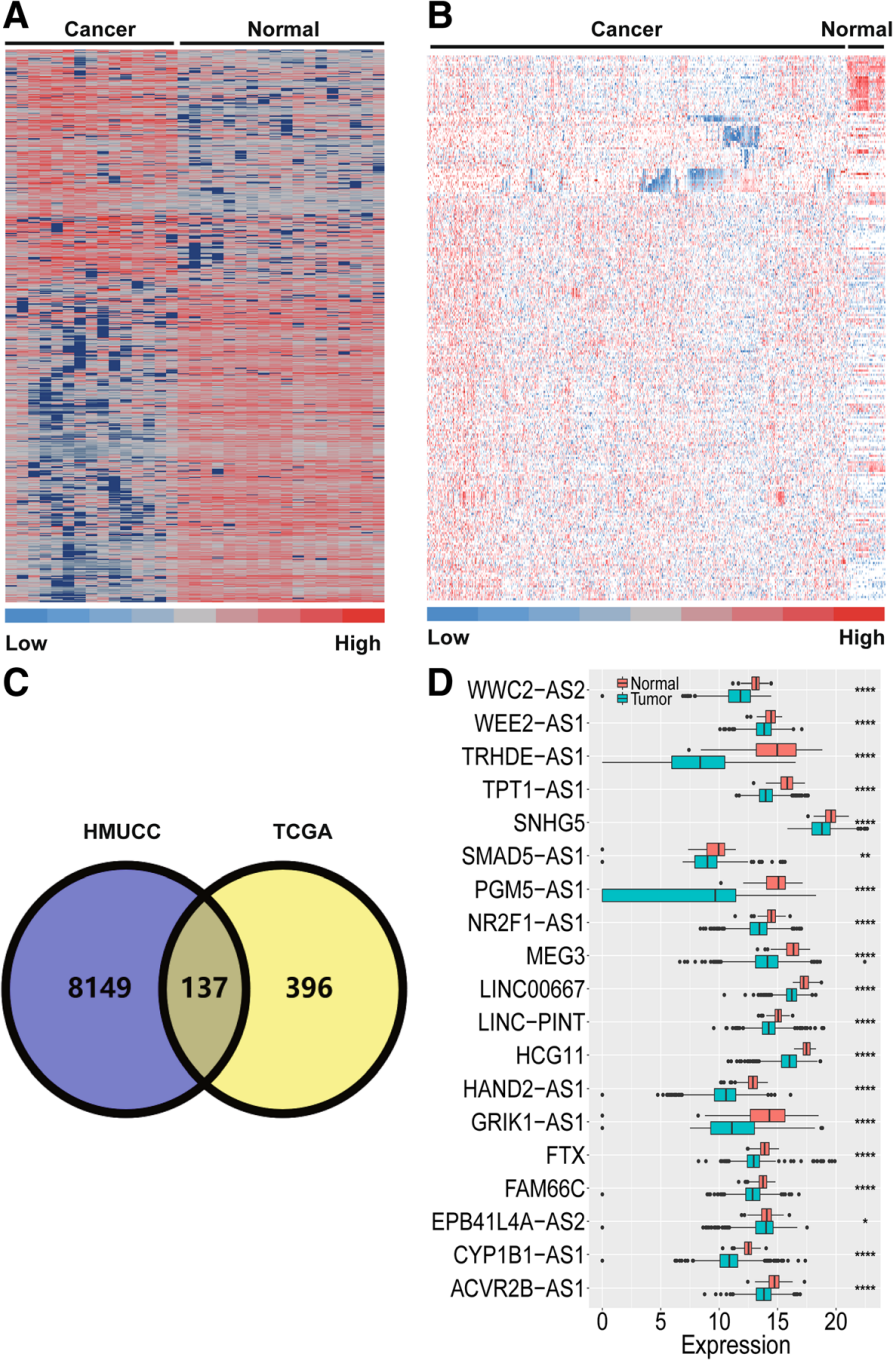
Discovery and validation of the TSLNR landscape in breast cancer. **a** Hierarchical clustering of the genes differentially expressed in breast cancer relative to normal tissue in the HMUCC cohort (*N* = 33) (∣FC∣ > 1.5, *P* < 0.05). **b** Hierarchical clustering of the genes differentially expressed in breast cancer relative to normal tissue in the TCGA cohort (*N* = 1105) (∣FC∣ > 1.5, *P* < 0.05). The red to blue color gradient indicates high to low expression levels, respectively. **c** Overlapping lncRNAs with downregulated expression in the HMUCC and TCGA breast cancer cohorts identified via Venny online software analysis (http://bioinfogp.cnb.csic.es/tools/venny/). **d** TSLNRs validated in the TCGA breast cancer cohort (∣FC∣ > 1.5, *P* < 0.001). ****P < 0. 0001; **P < 0. 01; *P < 0.05; NS: no significance

First, 33 breast tissue samples in the HMUCC cohort including 15 paired cancer tissue and corresponding adjacent normal tissue samples and 3 completely normal breast tissue samples acquired during mammoplasty, were subjected to whole transcriptome sequencing. The aberrant differential expression of lncRNAs among these tissues was obtained (Fig. 1a and Additional file 1: Table S1). Moreover, the differential expression of lncRNAs from breast cancer patients in the TCGA portal was also investigated in detail (Fig. 1b and Additional file 1: Table S2). Next, the overlapping differentially expressed lncRNAs between the HMUCC cohort and the TCGA cohort were investigated, and 137 differentially expressed lncRNAs were identified in both cohorts (Fig. 1c and Additional file 1: Table S3). Finally, the filter criteria for differentially downregulated lncRNAs was further restricted in the HMUCC cohort, and 19 differentially downregulated lncRNAs were identified and then validated in the TCGA breast cancer cohort (Fig. 1d and Additional file 1: Table S4). Thus, we defined these 19 differentially downregulated lncRNAs as TSLNRs.

### Extension validation of TSLNR expression in the pancancer cohort

**Fig. 2:**
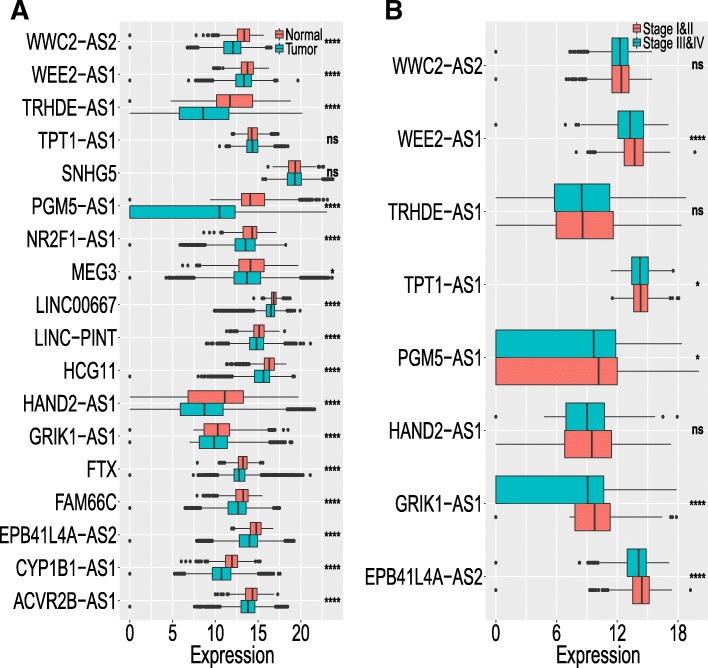
Extension validation of TSLNR expression in the pancancer cohort. **a** TSLNRs screened from the HMUCC and TCGA breast cancer cohorts were revalidated in the TCGA pancancer data, which included 33 cancer types and 12,839 patients. **b** TSLNRs that were expressed at significantly higher levels in the patients with early TNM stage (stage I & II) disease than in those with advanced stage (stage III & IV) disease in the pancancer data from TCGA. ****P < 0. 0001; *P < 0.05; NS: no significance

Next, the expression of the 19 TSLNRs identified above were revalidated in the HMUCC and TCGA pancancer data that included 33 cancer types and 12,839 patients. In the HMUCC dataset, 11 lncRNAs (WWC2-AS2, PGM5-AS1, MEG3, LINC00667, LINC-PINT, HAND2-AS1, FTX, FAM66C, EPB41L4A-AS2, CYP1B1-AS1 and ACVR2B-AS1) exhibited downregulated expression in breast cancer tissue compared with normal tissue (Additional file 2: Figure S1). In the TCGA dataset, 16 lncRNAs (WWC2-AS2, WEE2-AS1, TRHDE-AS1, PGM5-AS1, NR2F1-AS1, MEG3, LINC00667, LINC-PINT, HCG11, HAND2-AS1, GRIK1-AS1, FTX, FAM66C, EPB41L4A-AS2, CYP1B1-AS1 and ACVR2B-AS1) exhibited significantly downregulated expression in cancer tissue compared to normal tissue, consistent with the breast cancer expression model (Fig. 2a). Genetic alteration was also examined for these lncRNAs and there was no significant amplification or deep deletion in genome in breast cancer (Additional file 1: Table S4; Additional file 2: Figure S2). Furthermore, the relationships between the expression of these lncRNAs and the TNM stages of the patients in the pancancer data from the TCGA cohort were examined, and the expression of eight lncRNAs (WWC2-AS2, WEE2-AS1, TRHDE-AS1, TPT1-AS1, PGM5-AS1, HAND2-AS1, GRIK1-AS1 and EPB41L4A-AS2) was negatively related to advanced stages of cancer, but the relationships of three lncRNAs (WWC2-AS2, TRHDE-AS1 and HAND2-AS1) were not statistically significant (Fig. 2b). Five of the lncRNAs (WEE2-AS1, TPT1-AS1, PGM5-AS1, GRIK1-AS1 and EPB41L4A-AS2) were expressed at significantly higher levels in the patients with early TNM stage (stage I & II) disease than in those with advanced stage (stage III & IV) disease in the pancancer data from TCGA (Fig. 2c).

### TSLNR expression correlated with favorable prognosis in the breast cancer and pancancer datasets

Whether the TSLNRs discovered in this study are associated with the prognosis of breast cancer patients was unknown. Thus, we examined the relationships between the expression of the TSLNRs and OS or DFS in breast cancer patients in detail. The results showed that high expression of ACVR2B-AS1 or WEE2-AS1 was associated with favorable OS in breast cancer patients in the TCGA cohort (Additional file 2: Figure S3). Moreover, high expression of ACVR2B-AS1, WEE2-AS1, LINC-PINT or HAND2-AS1 was associated with favorable DFS in breast cancer patients in the TCGA cohort (Additional file 2: Figure S3). For the other lncRNAs, high expression of CYP1B1-AS1, LINC-PINT, LINC00667 or GRIK1-AS1 was associated with favorable OS, but the improvements were not statistically significant (Additional file 2: Figure S4). Finally, high expression of CYP1B1-AS1, FAM66C or GRIK1-AS1 was associated with favorable OS, but the improvements were not statistically significant (Additional file 2: Figure S3).

**Fig. 3:**
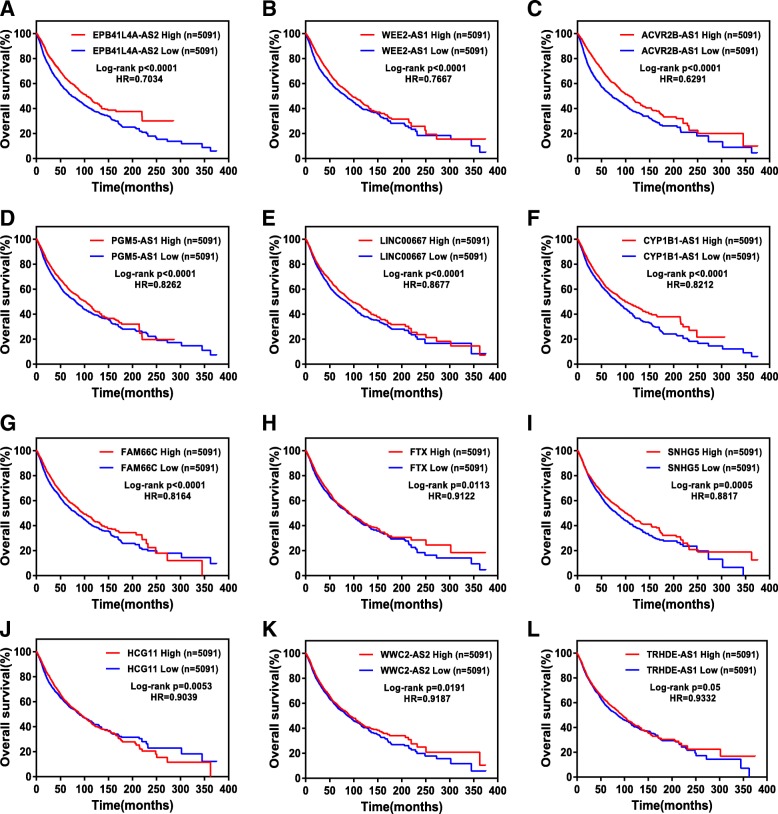
TSLNR expression correlated with favorable overall survival in the pancancer cohort. (**a**-**l**) The patients with high expression (*N* = 5091) of TSLNRs (EPB41L4A-AS2 (**a**), WEE2-AS1 (**b**), ACVR2B-AS1 (**c**), PGM5-AS1 (**d**), LINC00667 (**e**), CYP1B1-AS1 (**f**), FAM66C (**g**), FTX (**h**), SNHG5 (**i**), HCG11 (**j**), WWC2-AS2 (**k**) and TRHDE-AS1 (**l**)) had more favorable overall survival than those with low expression (*N* = 5091) in the pancancer cohort

**Fig. 4:**
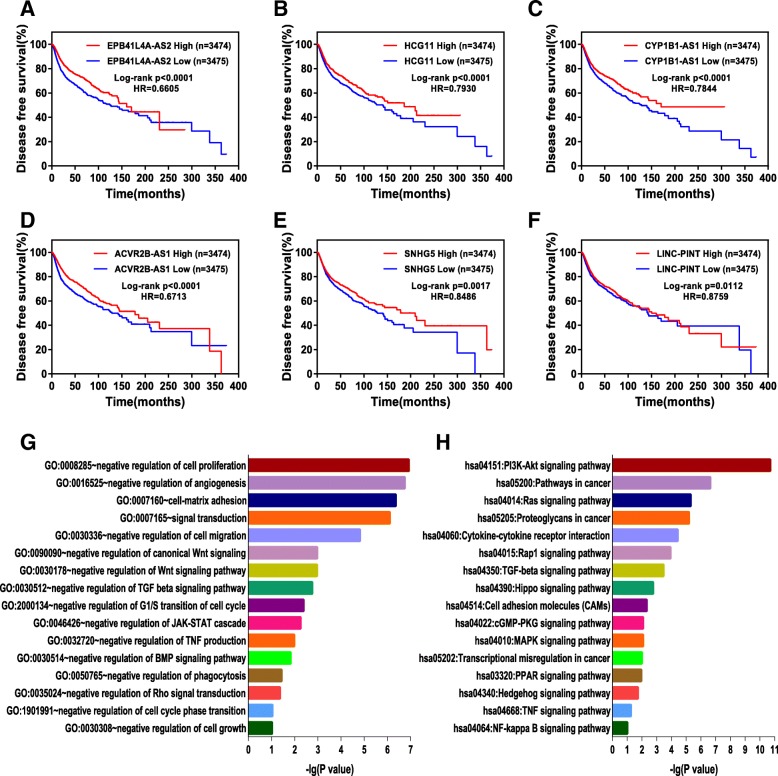
TSLNR expression correlated with favorable disease-free survival in the pancancer cohort, and the potential biological functions of the TSLNRs in breast cancer were evaluated. **a-f** The patients with high expression (*N* = 3474) of TSLNRs (EPB41L4A-AS2, HCG11, CYP1B1-AS1, ACVR2B-AS1, SNHG5 and LINC-PINT) had more favorable disease-free survival than those with low expression (*N* = 3475) in the pancancer cohort. **g** Gene ontology enrichment analysis was used to assess TSLNR-correlated genes obtained by guilt-by-association analysis. **h** KEGG analysis was used to assess TSLNR-correlated genes obtained by guilt-by-association analysis. The vertical axis represents the biological process or pathway category, and the horizontal axis represents the -log_10_ (*P* value) of the significant biological process or pathway

To validate the results obtained in the breast cancer cohorts, the TCGA pancancer data were applied to examine the relationship between TSLNR expression and OS or DFS. The results showed that high expression of EPB41L4A-AS2, WEE2-AS1, ACVR2B-AS1, PGM5-AS1, LINC00667, CYP1B1-AS1, FAM66C, FTX, SNHG5, HCG11, WWC2-AS2 or TRHDE-AS1 was associated with favorable OS in the patients in the pancancer data from TCGA (Fig. 3). Moreover, high expression of EPB41L4A-AS2, HCG11, CYP1B1-AS1, ACVR2B-AS1, SNHG5 or LINC-PINT was associated with favorable DFS in patients in this cohort (Fig. 4a-f).

### Potential biological functions of TSLNRs in breast cancer

To explore the potential biological functions of TSLNRs, guilt-by-association analysis was applied to perform the following analyses (Additional file 1: Table S5). TSLNRs may negatively regulate multiple tumor biological behaviors, including cell proliferation, angiogenesis, cell migration, cell-matrix adhesion, Wnt signaling transduction, mitotic cell cycle phase transition, JAK-STAT signaling transduction, tumor necrosis factor (TNF) production, BMP signaling transduction, cell adhesion mediated by integrin, cAMP biosynthesis, phagocytosis, Rho protein signal transduction, and platelet-derived growth factor receptor signaling transduction (Fig. 4g and Additional file 1: Table S5). The pathways involving TSLNRs were further examined. The results indicated that TSLNRs may be involved in several vital oncogenic signaling pathways, including the PI3K-Akt signaling pathway, the Ras signaling pathway, proteoglycans in cancer, cytokine-cytokine receptor interactions, the Rap1 signaling pathway, the TGF-beta signaling pathway, the Hippo signaling pathway, the cGMP-PKG signaling pathway, the MAPK signaling pathway, the PPAR signaling pathway, the Hedgehog signaling pathway, the TNF signaling pathway, the NF-kappa B signaling pathway (Fig. 4h and Additional file 1: Table S5).

### Epigenetic modification leads to the downregulation of TSLNR expression in breast cancer

**Fig. 5:**
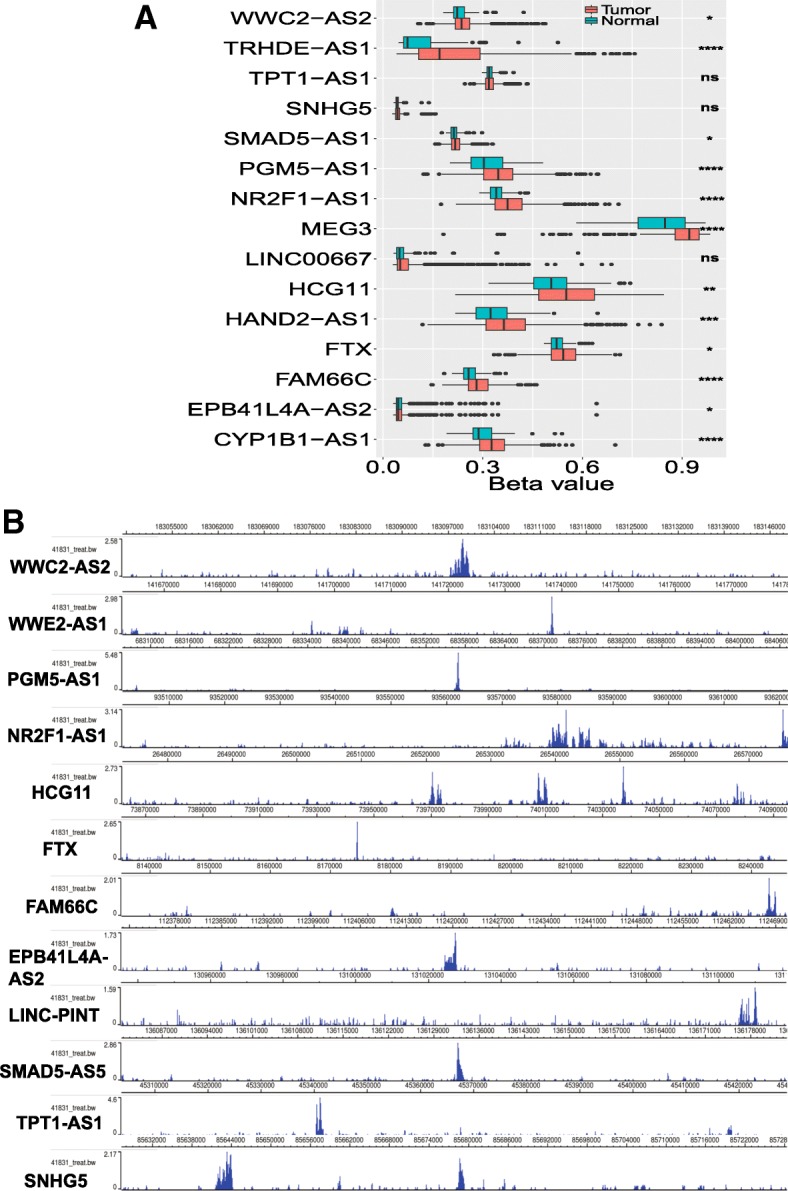
Epigenetic modification leads to downregulation of TSLNR expression in breast cancer. **a** TSLNRs (WWC2-AS2, TRHDE-AS1, SMAD1-AS1, PGM5-AS1, NR2F1-AS1, MEG3, HCG11, HAND2-AS1, FTX, FAM66C, EPB41L4A-AS2 and CYP1B1-AS1) exhibited higher levels of DNA methylation in cancer tissue than normal tissue in the Illumina Infinium HumanMethylation450 Beadchip data analysis of the TCGA breast cancer cohort. **b** TSLNRs (WWC2-AS2, WEE2-AS1, PGM5-AS1, NR2F1-AS1, LINC-PINT, HCG11, FTX, FAM66C, EPB41L4A-AS2, SMAD5-AS1, TPT1-AS1 and SNHG5) showed a significant H3K27me3 enrichment peak at each TSLNR locus in the MDA-MB-231 cell data obtained from the ENCODE database

Why is the expression of these TSLNRs downregulated in both the human breast cancer data and the pancancer data? The Illumina Infinium HumanMethylation450 Beadchip data in the TCGA portal was downloaded and investigated carefully to explore the beta value differences between cancer tissues and normal tissues for each TSLNR locus. The results showed that 12 TSLNR genome loci (those of WWC2-AS2, TRHDE-AS1, SMAD1-AS1, PGM5-AS1, NR2F1-AS1, MEG3, HCG11, HAND2-AS1, FTX, FAM66C, EPB41L4A-AS2 and CYP1B1-AS1) exhibited higher levels of DNA methylation in cancer tissues than normal tissues (Fig. 5a). Thus, the low expression of TSLNRs, at least in part, may be the result of the hypermethylation of each TSLNR genome locus in breast cancer.

Histone methylation modification was next investigated as it is usually accompanied by DNA methylation. The H3K27me3 enrichment peak for each TSLNR genome locus in MDA-MB-231 cells was investigated in the ENCODE data. As expected, 12 TSLNRs (WWC2-AS2, WEE2-AS1, PGM5-AS1, NR2F1-AS1, LINC-PINT, HCG11, FTX, FAM66C, EPB41L4A-AS2, SMAD5-AS1, TPT1-AS1 and SNHG5) showed significant H3K27me3 enrichment peaks at the corresponding TSLNR locus (Fig. 5b). Thus, the H3K27me3 histone methylation modification may also lead to the low expression of TSLNRs in breast cancer.

**Fig. 6:**
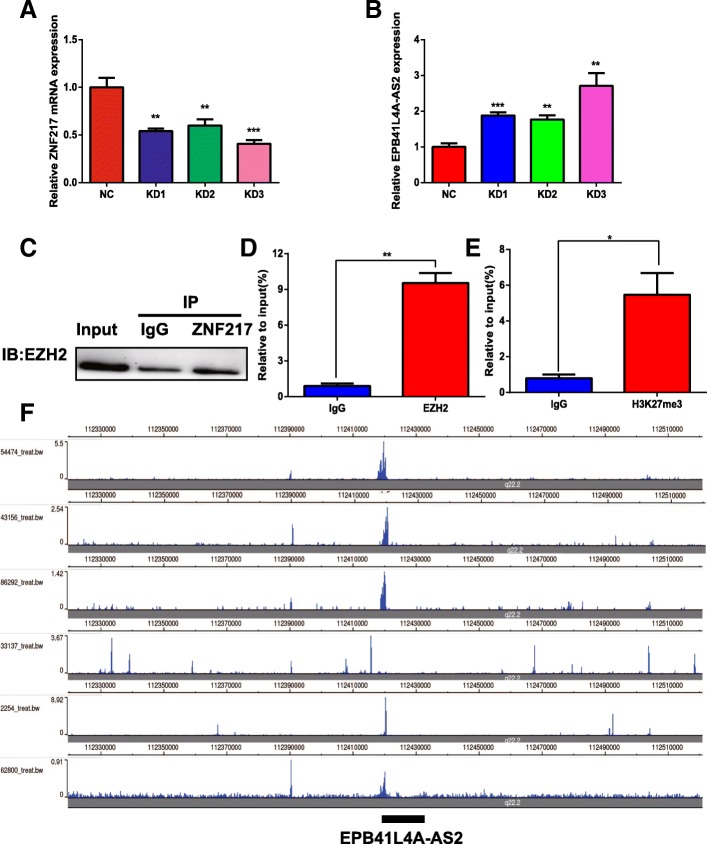
ZNF217-EZH2-H3K27me3 axis epigenetically suppresses the expression of EPB41L4A-AS2 in breast cancer. **a** The ZNF217 knockdown efficiencies of three siRNAs were examined. **b** The expression of EPB41L4A-AS2 was upregulated when ZNF217 expression was knocked down in MDA-MB-231 cells. **c** A Co-IP assay showed that ZNF217 can directly bind to EZH2. **d** ChIP- PCR showed that EZH2 could bind to the promoter region of EPB41L4A-AS2. **e** ChIP-PCR showed that H3K27me3 could bind to the promoter region of EPB41L4A-AS2. **f** A significant H3K27me3 enrichment peak occurred at the EPB41L4A-AS2 locus in the breast cancer cell data from ENCODE. ***P < 0. 001; **P < 0. 01; *P < 0.05. Data represent at least three independent experiments

Next, EPB41L4A-AS2 was selected to validate the histone methylation modification model, as we first reported the potential function of EPB41L4A-AS2 in human cancer [16], and an obvious H3K27me3 enrichment peak at the EPB41L4A-AS2 locus could be observed in MDA-MB-231 cells (Fig. 5b). ZNF217 has been reported to be a marker of poor prognosis in breast cancer that drives epithelial-mesenchymal transition and invasion by recruiting EZH2 to its target genes, which are marked with an H3K27me3 enrichment peak [35, 36]. Thus, we hypothesized that EPB41L4A-AS2 could be regulated by this model. Initially, the expression of EPB41L4A-AS2 was upregulated in MDA-MB-231 breast cancer cells with the knockdown of ZHF217 expression (Fig. 6a and b). Moreover, EPB41L4A-AS2 expression was also found to be downregulated in MDA-MB-231 breast cancer cells overexpressing ZNF217 in the GEO dataset GSE35511 (Additional file 2: Figure S5). Next, a Co-IP assay showed that ZNF217 can directly bind to EZH2 (Fig. 6c). ChIP followed by PCR showed that EZH2 could bind to the promoter region of EPB41L4A-AS2 (Fig. 6d). Furthermore, H3K27me3 was also found to be enriched in the EPB41L4A-AS2 locus via ChIP (Fig. 6e). Finally, to extend our results, the data from ENCODE were examined again, and the results showed that a significant H3K27me3 enrichment peak could be observed at the EPB41L4A-AS2 locus in breast cancer cells (Fig. 6f). Thus, the ZNF217-EZH2-H3K27me3 axis epigenetically suppresses the expression of EPB41L4A-AS2 in breast cancer.

### Potential biological functions of EPB41L4A-AS2 in breast cancer

**Fig. 7:**
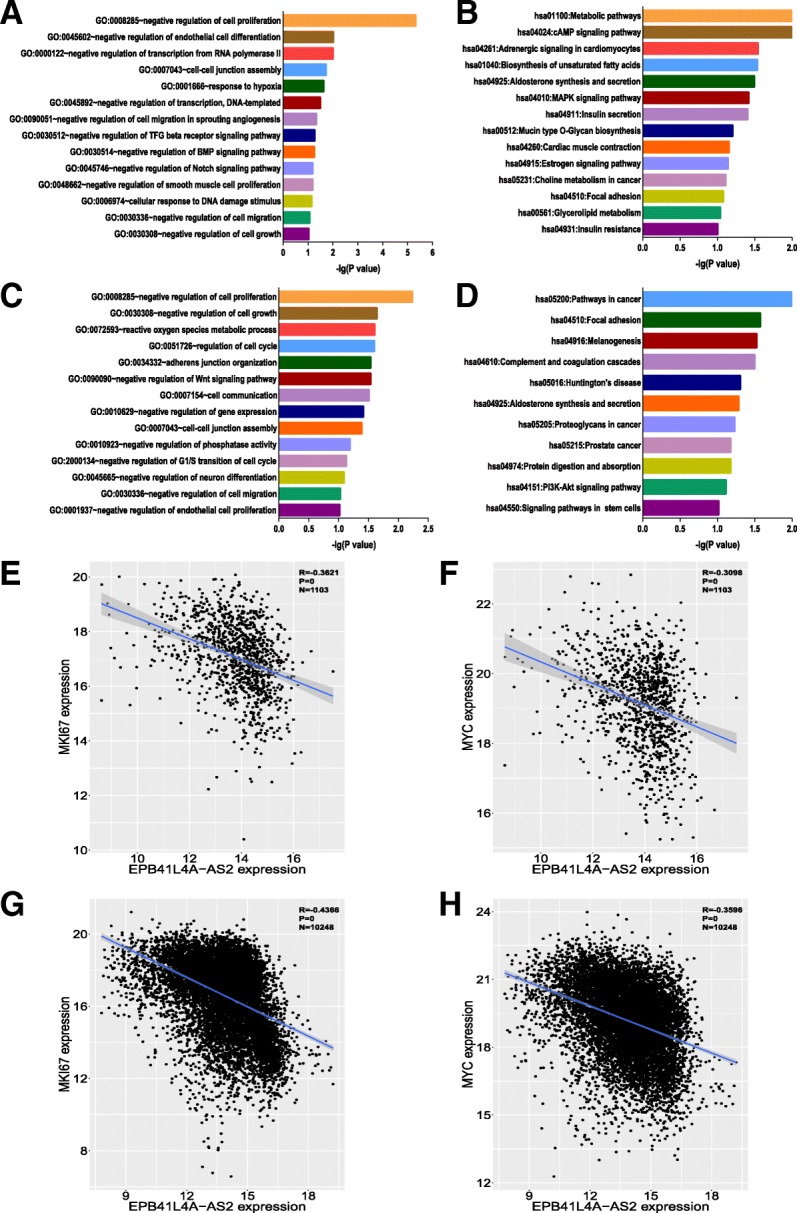
Potential biological functions of EPB41L4A-AS2 in breast cancer. **a** Gene ontology enrichment analysis of EPB41L4A-AS2-correlated genes obtained by guilt-by-association analysis of the breast cancer data in TCGA. **b** KEGG analysis of EPB41L4A-AS2-correlated genes obtained by guilt-by-association analysis of the breast cancer data in TCGA. **c** Gene ontology enrichment analysis of EPB41L4A-AS2-correlated genes obtained by guilt-by-association analysis of the pancancer data in TCGA. **d** KEGG analysis of EPB41L4A-AS2-correlated genes obtained by guilt-by-association analysis of the pancancer data in TCGA. The vertical axis represents the biological process or pathway category, and the horizontal axis represents the -log_10_ (*P* value) of the significant biological process or pathway. **e** Correlation between the expression of EPB41L4A-AS2 and the expression of MKI67 in the breast cancer data in TCGA (*N* = 1103). **f** Correlation between the expression of EPB41L4A-AS2 and the expression of MYC in the breast cancer data in TCGA (N = 1103). **g** Correlation between the expression of EPB41L4A-AS2 and the expression of MKI67 in the pancancer data in TCGA (*N* = 10,248). **h** Correlation between the expression of EPB41L4A-AS2 and the expression of MYC in the pancancer data in TCGA (*N* = 10,248)

A guilt-by-association approach was applied to analyze the potential biological functions of EPB41L4A-AS2 in breast cancer with the TCGA breast cancer data (Additional file 1: Table S6). EPB41L4A-AS2 may negatively regulate multiple biological functions involved in tumors, including cell proliferation, cell migration, TGF-beta signaling transduction, BMP signaling transduction and Notch signaling transduction (Fig. 7a). KEGG analysis showed that EPB41L4A-AS2 may participate in multiple oncogenic signaling pathways, including metabolic pathways, the cAMP signaling pathway, the MAPK signaling pathway, and the estrogen signaling pathway (Fig. 7b). Finally, to extend our results, the potential biological functions of EPB41L4A-AS2 in the pancancer dataset were investigated. The results indicated that EPB41L4A-AS2 may be involved in the key biological functions and pathways of carcinogenesis and tumor progression, and these results agreed with the results of the breast cancer analysis (Fig. 7c and d and Additional file 1: Table S7). Moreover, pharmacological network analysis indicates that EPB41L4A-AS2 may be involved in the regulation of paclitaxel activity in breast cancer (Additional file 2: Figure S6), which is in agreement with the predicted roles of EPB41L4A-AS2 in junction assembly and mitosis (Additional file 1: Table S6).

As EPB41L4A-AS2 was predicted to suppress cancer cell proliferation both in the breast cancer and pancancer TCGA data, we next wondered whether EPB41L4A-AS2 expression was negatively correlated with the expression of classic oncogenes in human cancer. As MKI67 and MYC are two famous driver oncogenes in carcinogenesis and tumor progression [37, 38], the relationships between the expression levels of EPB41L4A-AS2, MKI67 and MYC were next explored. As expected, the expression of EPB41L4A-AS2 was negatively correlated with the expression of MKI67 and MYC both in breast cancer patients and the patients in the pancancer cohort (Fig. 7e-h). Collectively, these results indicate that EPB41L4A-AS2 is negatively related to the malignant behaviors of cancer and negatively correlated with oncogene expression.

### EPB41L4A-AS2 inhibited cell proliferation, migration and invasion in breast cancer

**Fig. 8:**
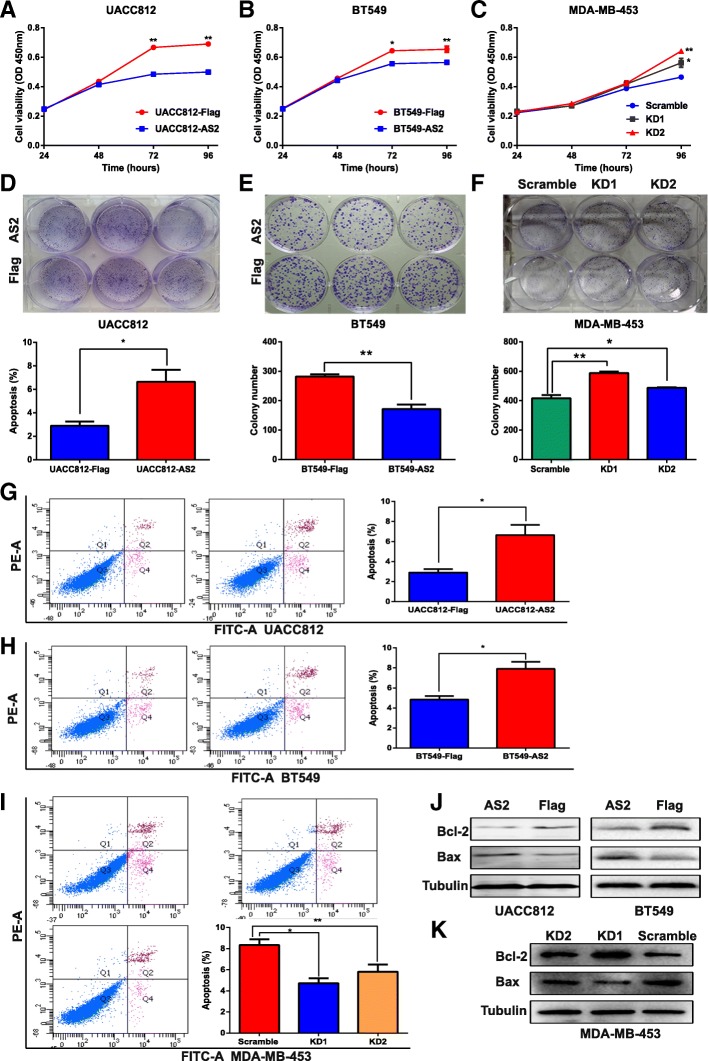
EPB41L4A-AS2 inhibits cell proliferation in breast cancer. **a** and **b** Compared with the overexpression of a control, the overexpression of EPB41L4A-AS2 significantly inhibited cell proliferation in UACC812 and BT549 cells, as measured by CCK-8 assays. **c** The knockdown of EPB41L4A-AS2 expression promoted cell proliferation in MDA-MB-453 cells, as measured by CCK-8 assays. **de** The overexpression of EPB41L4A-AS2 inhibited clone formation in UACC812 and BT549 cells. **f** The knockdown of EPB41L4A-AS2 expression increased clone formation in MDA-MB-453 cells. **g** and **h** Compared with the overexpression of a control, the overexpression of EPB41L4A-AS2 significantly promoted cell apoptosis in UACC812 and BT549 cells, as measured by flow cytometry detection. **i** The knockdown of EPB41L4A-AS2 expression suppressed cell apoptosis in MDA-MB-453 cells, as measured by flow cytometry detection. **j** Bcl-2 expression was reduced, and Bax expression was increased in UACC812 and BT549 cells overexpressing EPB41L4A-AS2. **k** The knockdown of EPB41L4A-AS2 expression increased Bcl-2 expression and reduced Bax expression in MDA-MB-453 cells. **P < 0. 01; *P < 0.05. Data represent at least three independent experiments

First, the expression level of EPB41L4A-AS2 was examined in eight breast cancer cell lines (Additional file 2: Figure S7a). UACC812, BT549 and MDA-MB-453 cells were ultimately selected to perform the following experiments as UACC812 and BT549 cells have low expression of EPB41L4A-AS2 and MDA-MD-453 has high expression of EPB41L4A-AS2. Next, the overexpression and knockdown efficiencies were examined in these three cell lines (Additional file 2: Figure S7b-d). Compared with Flag (control) overexpression, the overexpression of EPB41L4A-AS2 inhibited cell proliferation significantly in UACC812 and BT549 cells (Fig. 8a and b). In contrast, knocking down EPB41L4A-AS2 expression promoted cell proliferation in MDA-MB-453 cells (Fig. 8c). Furthermore, similar results that showed that the overexpression of EPB41L4A-AS2 inhibited clone formation while knocking down EPB41L4A-AS2 expression increased clone formation were observed in clone formation assays with the above three cell lines (Fig. 8d-f).

**Fig. 9:**
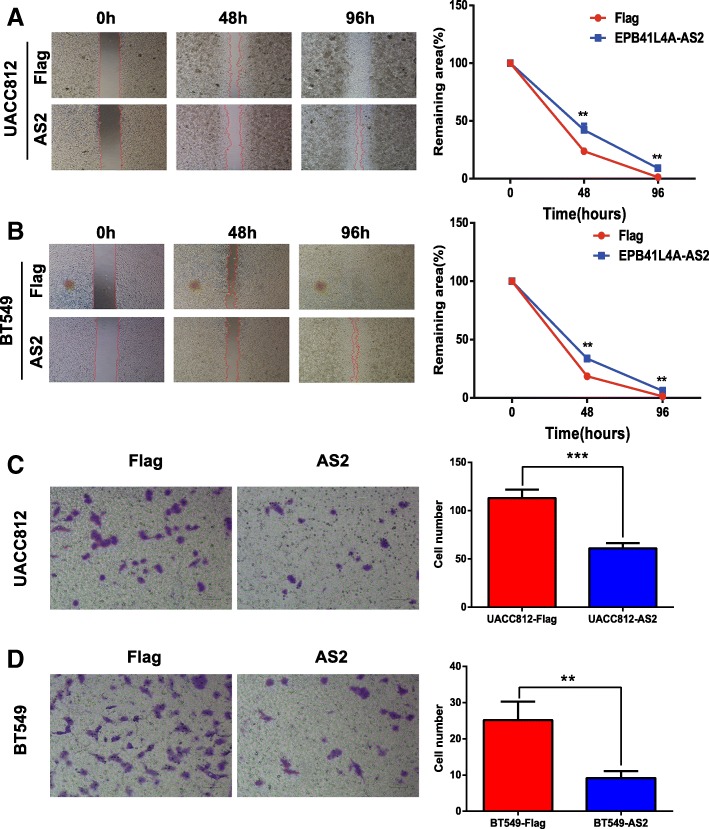
EPB41L4A-AS2 inhibits cell migration and invasion in breast cancer. **a-b** The overexpression of EPB41L4A-AS2 reduced cell migration in UACC812 (**a**) and BT549 cells (**b**), as measured by wound healing assays. **c**-**d** The overexpression of EPB41L4A-AS2 reduced cell invasion in UACC812 (**c**) and BT549 cells (**d**), as measured by transwell assays. ***P < 0. 001; **P < 0. 01. Data represent at least three independent experiments

With regards to cell apoptosis, overexpressing EPB41L4A-AS2 triggered more cell apoptosis, while knocking down EPB41L4A-AS2 suppressed cell apoptosis in the three cell lines, as determined by flow cytometry analysis (Fig. 8g-i). Finally, cell apoptosis-associated markers were also investigated. Bcl-2 expression was reduced, and Bax expression was increased in the UACC812 and BT549 cells overexpressing EPB41L4A-AS2 (Fig. 8j). In MDA-MB-453 cells, knocking down EPB41L4A-AS2 expression increased Bcl-2 expression and reduced Bax expression (Fig. 8k). As EPB41L4A-AS2 was shown to inhibit the proliferation of breast cancer cells, we then investigated whether it could suppress cell migration and invasion in the abovementioned cell lines via wound healing and transwell assays. As expected, the overexpression of EPB41L4A-AS2 reduced migration (Fig. 9a) and invasion in UACC812 and BT549 cells (Fig. 9b). In addition to EPB41L4A-AS2, three other lncRNAs, MEG3, WEE2-AS1 and HAND2-AS1, were also applied to examine their potential tumor suppressor roles in breast cancer. As expected, the overexpression of each lncRNA inhibited clone formation in UACC812 cells (Additional file 2: Figure S8). Moreover, the overexpression of each lncRNA inhibited cell viability in UACC812 cells, matching the EPB41L4A-AS2 results (Additional file 2: Figure S8).

### EPB41L4A-AS2 functions as a tumor suppressor via the upregulation of RARRES1 expression

**Fig. 10:**
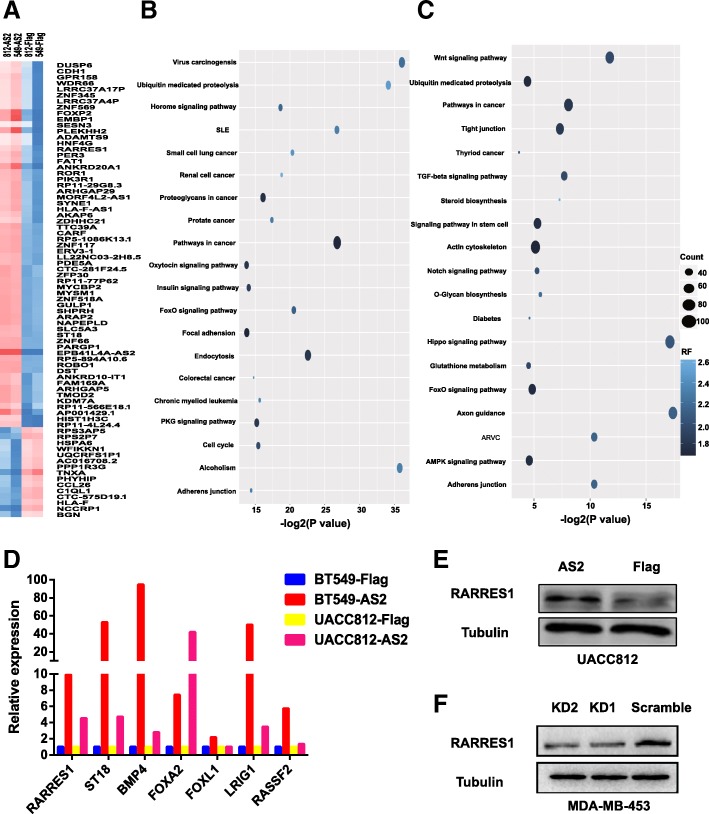
EPB41L4A-AS2 functions as a tumor suppressor via the upregulation of RARRES1 expression. **a** Hierarchical clustering of the genes differentially expressed between the EPB41L4A-AS2 overexpression and control groups of UACC812 and BT549 cells. The red to blue color gradient indicates high to low expression levels, respectively. **b** GO analysis of the genes differentially expressed between the EPB41L4A-AS2 overexpression and control group. **c** KEGG analysis of the genes differentially expressed between the EPB41L4A-AS2 overexpression and control groups. The vertical axis represents the biological process or pathway category, and the horizontal axis represents the -log_2_ (*P* value) of the significant biological process or pathway. **d** Validation of seven genes differentially expressed (RARRES1, ST18, BMP4, FOXA2, FOXL1, LRIG1 and RASSF1) in UACC812 and BT549 breast cancer cell lines via PCR array. **e** EPB41L4A-AS2 overexpression increased RARRES1 protein expression in UACC812 cells. **f** The knockdown of EPB41L4A-AS2 expression reduced RARRES1 protein expression in MDA-MB-453 cells. Data represent at least three independent experiments

Whole transcriptome sequencing of the UACC812 and BT549 breast cancer cell lines with or without EPB41L4A-AS2 overexpression was performed to identify potential target genes of EPB41L4A-AS2. The differentially expressed genes were measured and identified in different groups (Fig. 10a and Additional file 1: Table S8). Based on the KEGG analysis of these differentially expressed genes, EPB41L4A-AS2 may be involved in multiple cancer-associated pathways such as the hormone signaling pathway, FoxO signaling pathway and PKG signaling pathway in UACC812 cells (Fig. 10b). According to the KEGG analysis, EPB41L4A-AS2 may also be involved with the proteoglycans in the cancer-related Wnt signaling pathway, and in the TGF-beta signaling pathway, Notch signaling pathway, Hippo signaling pathway and AMPK signaling pathway in BT549 cells (Fig. 10c), and these results, to some extent, were consistent with those of our previous analysis (Fig. 7a-d). Next, a PCR array was applied to verify the differentially expressed genes identified by transcriptome sequencing, and we found that the expression levels of seven genes (RARRES1, ST18, BMP4, FOXA2, FOXL1, LRIG1 and RASSF1), which have all been reported to be tumor suppressors in breast cancer, were upregulated significantly with the overexpression of EPB41L4A-AS2 in the UACC812 and BT549 breast cancer cell lines (Fig. 10e). Moreover, RARRES1 protein expression was increased in the UACC812 cells overexpressing EPB41L4A-AS2 (Fig. 10e) and decreased in the MDA-MB-453 cells with knocked down EPB41L4A-AS2 expression (Fig. 10f). Thus, EPB41L4A-AS2 functions as a tumor suppressor at least in part via the upregulation of RARRES1 expression in breast cancer.

## Discussion

To our knowledge, no study has focused on the systematic identification of TSLNRs in human diseases, especially in human breast cancer. Moreover, few lncRNAs that function as tumor suppressors and are associated with a favorable prognosis have been identified in breast cancer. Thus, to solve this issue, TSLNRs were identified comprehensively and validated by whole transcriptome sequencing and analysis of TCGA breast cancer data via bioinformatic approaches and biological experiments in this study. We defined the identified lncRNAs as TSLNRs, enlarging the landscape of the functional lncRNA category. The levels of these TSLNRs are downregulated in cancer tissues compared with normal tissues. In addition, they were found to be correlated with a favorable prognosis in breast cancer patients and the patients in the pancancer cohort. Moreover, these TSLNRs are regulated by epigenetic modification, including DNA methylation and histone methylation modification. Finally, EPB41L4A-AS2, one of the verified TSLNRs, was selected to validate our novel findings, and it’s the tumor suppressor roles of EPB41L4A-AS2 in breast cancer were examined. In addition to mediating the DNA methylation regulation of EPB41L4A-AS2, ZNF217 recruited EZH2 to the EPB41L4A-AS2 locus and suppressed the expression of EPB41L4A-AS2 by epigenetically increasing H3K27me3 enrichment. Moreover, EPB41L4A-AS2 inhibited breast cancer cell proliferation, migration and invasion and induced cell apoptosis. To some extent, EPB41L4A-AS2 functioned as a tumor suppressor by upregulating the expression of RARRES1 in breast cancer.

Among the 19 TSLNRs in this study, only eight (EPB41L4A-AS2, MEG3, LINC-PINT, FTX, HCG11, HAND2-AS1, SNHG5 and TPT1-AS1) have been reported previously to be associated with malignancy, and the potential functions of the other 11 lncRNAs in human cancer remain a mystery. We were the first to report that EPB41L4A-AS2 inhibited tumor proliferation and was associated with favorable prognoses in breast cancer and other solid tumors [16]. Moreover, EPB41L4A-AS2 was also reported to inhibit proliferation and invasion and promote cell apoptosis in non-small cell lung cancer [39], in accordance with our results. Among the other identified lncRNAs, MEG3 was reported to suppress cell proliferation, invasion, and angiogenesis through the AKT pathway or the transcriptional activity of p53 in breast cancer [23–25, 40, 41]. LINC-PINT, which is regulated by p53, inhibits tumor cell invasion through a highly conserved sequence element [42, 43]. FTX was reported to inhibit hepatocellular carcinoma proliferation and metastasis by binding MCM2 and miR-374a [44]. In addition, HCG11 was found to suppress apoptosis via MAPK signaling transduction in hepatocellular carcinoma, and the downregulation of HCG11 expression predicts a poor prognosis in prostate cancer [45, 46]. A study also revealed that SNHG5 suppresses gastric cancer progression by trapping MTA2 in the cytosol [47]. In contrast, other studies have indicated that SNHG5 functions as an oncogene in colorectal cancer, osteosarcoma, hepatocellular cancer, bladder cancer and gastric cancer [48–52]. TPT1-AS1 was reported to promote cell growth and metastasis in cervical cancer by acting as a sponge for miR-324-5p [53]. However, our study suggested that TPT1-AS1 may be a tumor suppressor in breast cancer. The functional diversity of SNHG5 and TPT1-AS1 in various malignancies may be caused by tumor heterogeneity, and subsequent research is necessary to reveal the potential roles of these two lncRNAs.

This study addresses three distinct merits that are not evident in other studies. First, a panel of TSLNRs was identified via whole transcriptome sequencing and TCGA data analysis, thus providing a more efficient research methodology and opening research into this field in human cancer. Hence, the drawbacks of a single lncRNA-focused study can be avoided. Second, TSLNRs were comprehensively further validated in the TCGA pancancer data containing 33 cancer types and 12,839 patients, increasing the study reliability and strengthening our theoretical basis. Third, DNA methylation and histone methylation modification led to the downregulation of TSLNR expression in breast cancer, further suggesting that DNA methylation inhibitors or histone methylation inhibitors may be effective agents in human cancer management. To date, DNA methyltransferase inhibitors have been reported to inhibit tumor growth and promote cell apoptosis in breast cancer, colon cancer, ovarian cancer, gastric cancer and human endometrial cancer [54–58]. Moreover, the histone methyltransferase inhibitor A-366 has been reported to suppress cancer cell viability by targeting G9a/GLP in leukemia [59]. In addition, 3-deazaneplanocin A, an inhibitor of the histone methyltransferase EZH2, inhibits the growth of non-small cell lung cancer cells [60]. Thus, the anticancer roles of epigenetic therapies targeting DNA methylation or histone methylation modification could be result from direct tumor suppressor functions and the reversed expression of TSLNRs, which also function as effective antitumor agents.

We acknowledge several limitations of our study. First, not all of the gene expression profiles in TCGA can be accessed. Thus, the TSLNR landscape in human breast cancer described in this study may not be sufficient. Second, the biological functions and detailed mechanisms of other TSLNRs were not elucidated. Further in-depth efforts will be needed to investigate TSLNRs other than EPB41L4A-AS2 and focus on molecular mechanisms. Third, pharmacological network analysis indicated that EPB41L4A-AS2 may be involved in the regulation of paclitaxel activity in breast cancer; however, this result should be examined and validated in our future investigation. Lastly, as gene therapy has its own complications, whether the upregulation of TSLNR expression with or without epigenetic therapy could be applied in cancer therapy requires more clinical trials to ensure safety and specificity.

## Conclusions

This work enlarges the functional landscape of known lncRNAs in human cancer and provides novel insights into their suppressive roles.

## Additional files

Additional file 1: **Table S1.** Differential expression of lncRNAs between breast cancer tissues and normal tissues in HMUCC cohort. **Table S2.** Differential expression of lncRNAs between breast cancer tissues and normal tissues in TCGA cohort. **Table S3.** Overlapped differential lncRNAs between HMUCC cohort and TCGA cohort. **Table S4.** Restricted filter criterial (FC < 0.833, *P* < 0.001) identified 19 differential downregulated lncRNAs in HMUCC cohort and chromosome position of these lncRNAs. **Table S5.** Guilt-by-association followed by GO and KEGG analyses of TSLCRs in breast cancer. **Table S6.** Guilt-by-association followed by GO and KEGG analyses of EPB41L4A-AS2 in breast cancer. **Table S7.** Guilt-by-association analyses followed by GO and KEGG of EPB41L4A-AS2 in pan-cancer. **Table S8.** Differential gene expression between EPB41L4A-AS2 overexpression groups and control groups in UACC812 and BT549 breast cancer cell lines. (ZIP 3607 kb)

Additional file 2: **Figure S1.** TSLNR expression in breast cancer samples and normal tissue samples in HMUCC. **Figure S2.** Genetic alteration was also examined for these lncRNAs in breast cancer data in TCGA. **Figure S3.** A&B Patients with high expression (*N* = 266) of TSLNRs (ACVR2B-AS1 and WEE2-AS1) had favorable OS than those with low expression (*N* = 266) in breast cancer in TCGA. C-F Patients with high expression (N = 266) of TSLNRs (ACVR2B-AS1, WEE2-AS1, LINC-PINT and HAND2-AS1) had favorable DFS than those with low expression (N = 266) in breast cancer in TCGA. **Figure S4.** A-D Patients with high expression (N = 266) of TSLNRs (CYP1B1-AS1, LINC-PINT, LINC00667 and GRIK1-AS1) had favorable OS than those with low expression (N = 266) in breast cancer in TCGA. E-G Patients with high expression (N = 266) of TSLNRs (CYP1B1-AS1, FAM66C and GRIK1-AS1) had favorable DFS than those with low expression (N = 266) in breast cancer in TCGA. **Figure S5.** EPB41L4A-AS2 was downregulated in MDA-MB-231 breast cancer cells with ZNF217 overexpression in GEO dataset GSE35511. **Figure S6.** A Overlapping genes of EPB41L4A-AS2 correlated genes and paclitaxel related genes in BETMAN-TCM. B KEGG pathway analysis for EPB41L4A-AS2 correlated genes in BETMAN-TCM. C GO analysis for EPB41L4A-AS2 correlated genes in BETMAN-TCM.D OMIM analysis for EPB41L4A-AS2 correlated genes in BETMAN-TCM.E Pharmacological network analysis indicates that EPB41L4A-AS2 may be involved in paclitaxel related process in breast cancer. F Pharmacological network analysis indicates that EPB41L4A-AS2 may be involved in crosstalk with paclitaxel related genes in breast cancer. **Figure S7.** A Expression of EPB41L4A-AS2 in breast cancer cell lines. B&C overexpression efficiency of EPB41L4A-AS2 in UACC812 and BT549 cells. D Knockdown efficiency of EPB41L4A-AS2 in MDA-MB-453 cells. **Figure S8.** A-C Overexpression of each lncRNA (MEG3, WEE2-AS1 and HAND2-AS1) inhibited clone formation in UACC812 cells. D-F Overexpression of each lncRNA (MEG3, WEE2-AS1 and HAND2-AS1) inhibited cell viability in UACC812 cells. (ZIP 19903 kb)

## Abbreviations

ENCODE: Encyclopedia of DNA Elements
GO: Gene ontology
HNSC: Head and neck squamous cell carcinoma
KEGG: Kyoto Encyclopedia of Genes and Genomes
SiRNA: Small interfering RNA
TCGA: The Cancer Genome Atlas
TSLNR: tumor suppressor long noncoding RNA
