# Supplementary figures and images for: Landscape of tumor suppressor long noncoding RNAs in breast cancer
Source: J Exp Clin Cancer Res. 2019 Feb 14;38:79. doi: 10.1186/s13046-019-1096-0 (PMC6376750; doi:10.1186/s13046-019-1096-0)

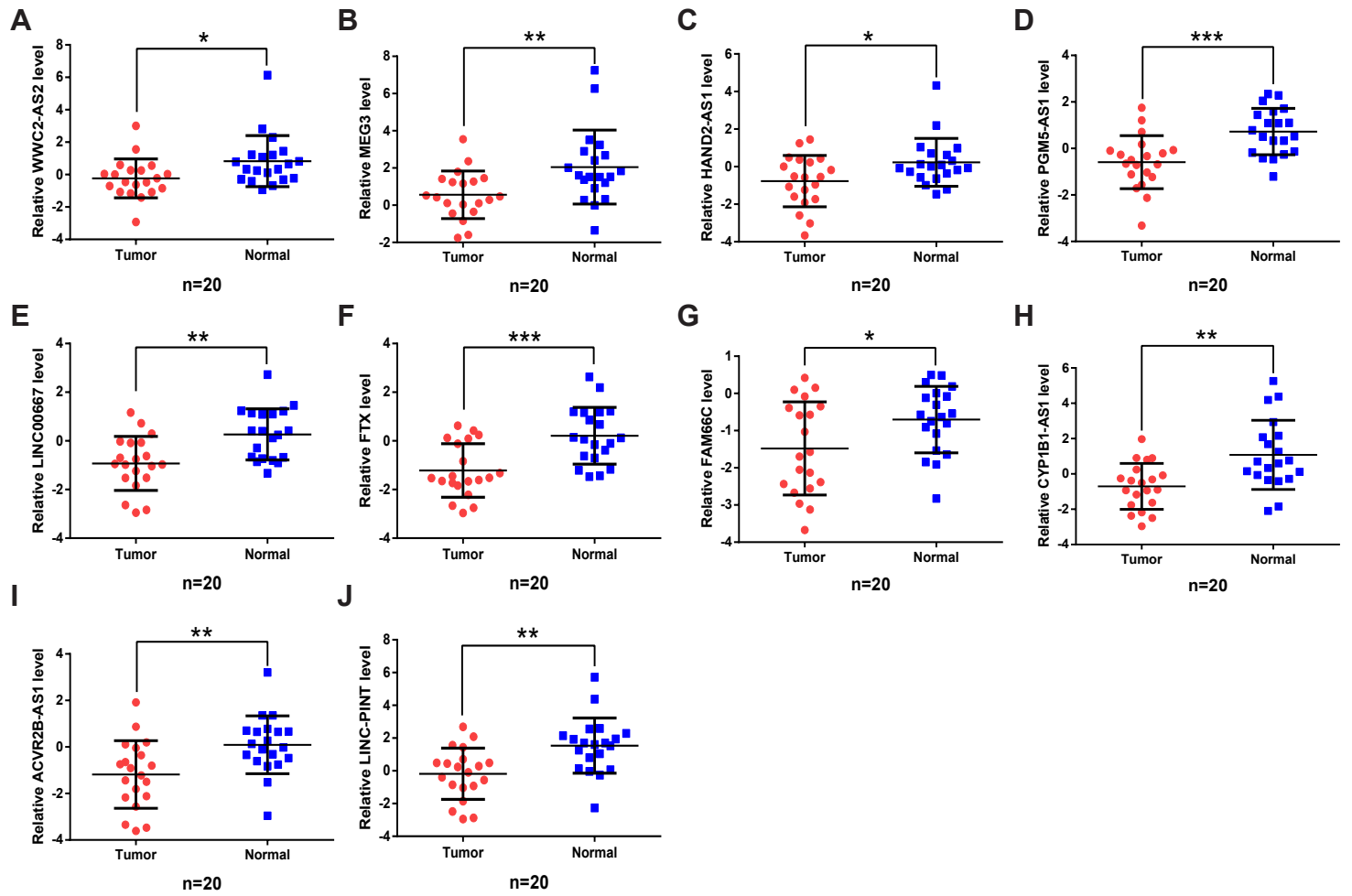

Supplement: Supplementary file 2 — Figure S1. TSLNR expression in breast cancer samples and normal tissue samples in HMUCC. Figure S2. Genetic alteration was also examined for these lncRNAs in breast cancer data in TCGA. Figure S3. A&B Patients with high expression (N = 266) of TSLNRs (ACVR2B-AS1 and WEE2-AS1) had favorable OS than those with low expression (N = 266) in breast cancer in TCGA. C-F Patients with high expression (N = 266) of TSLNRs (ACVR2B-AS1, WEE2-AS1, LINC-PINT and HAND2-AS1) had favorable DFS than those with low expression (N = 266) in breast cancer in TCGA. Figure S4. A-D Patients with high expression (N = 266) of TSLNRs (CYP1B1-AS1, LINC-PINT, LINC00667 and GRIK1-AS1) had favorable OS than those with low expression (N = 266) in breast cancer in TCGA. E-G Patients with high expression (N = 266) of TSLNRs (CYP1B1-AS1, FAM66C and GRIK1-AS1) had favorable DFS than those with low expression (N = 266) in breast cancer in TCGA. Figure S5. EPB41L4A-AS2 was downregulated in MDA-MB-231 breast cancer cells with ZNF217 overexpression in GEO dataset GSE35511. Figure S6. A Overlapping genes of EPB41L4A-AS2 correlated genes and paclitaxel related genes in BETMAN-TCM. B KEGG pathway analysis for EPB41L4A-AS2 correlated genes in BETMAN-TCM. C GO analysis for EPB41L4A-AS2 correlated genes in BETMAN-TCM.D OMIM analysis for EPB41L4A-AS2 correlated genes in BETMAN-TCM.E Pharmacological network analysis indicates that EPB41L4A-AS2 may be involved in paclitaxel related process in breast cancer. F Pharmacological network analysis indicates that EPB41L4A-AS2 may be involved in crosstalk with paclitaxel related genes in breast cancer. Figure S7. A Expression of EPB41L4A-AS2 in breast cancer cell lines. B&C overexpression efficiency of EPB41L4A-AS2 in UACC812 and BT549 cells. D Knockdown efficiency of EPB41L4A-AS2 in MDA-MB-453 cells. Figure S8. A-C Overexpression of each lncRNA (MEG3, WEE2-AS1 and HAND2-AS1) inhibited clone formation in UACC812 cells. D-F Overexpression of each lncRNA (MEG3, WEE2-AS1 and H [file 13046_2019_1096_MOESM2_ESM.zip › Figure S1.pdf]

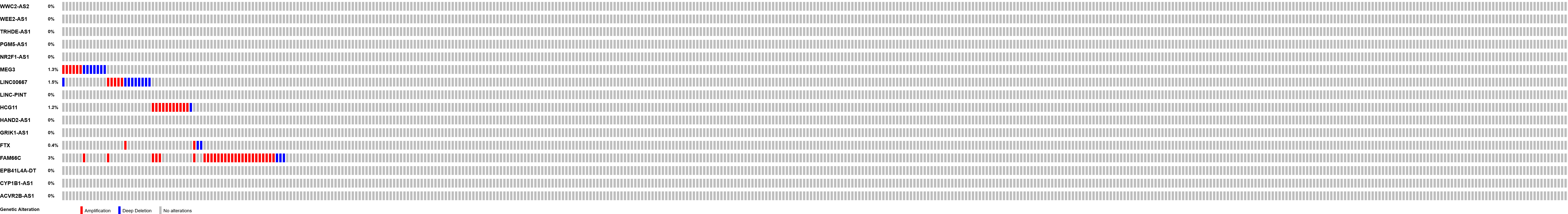

Supplement: Supplementary file 2 — Figure S1. TSLNR expression in breast cancer samples and normal tissue samples in HMUCC. Figure S2. Genetic alteration was also examined for these lncRNAs in breast cancer data in TCGA. Figure S3. A&B Patients with high expression (N = 266) of TSLNRs (ACVR2B-AS1 and WEE2-AS1) had favorable OS than those with low expression (N = 266) in breast cancer in TCGA. C-F Patients with high expression (N = 266) of TSLNRs (ACVR2B-AS1, WEE2-AS1, LINC-PINT and HAND2-AS1) had favorable DFS than those with low expression (N = 266) in breast cancer in TCGA. Figure S4. A-D Patients with high expression (N = 266) of TSLNRs (CYP1B1-AS1, LINC-PINT, LINC00667 and GRIK1-AS1) had favorable OS than those with low expression (N = 266) in breast cancer in TCGA. E-G Patients with high expression (N = 266) of TSLNRs (CYP1B1-AS1, FAM66C and GRIK1-AS1) had favorable DFS than those with low expression (N = 266) in breast cancer in TCGA. Figure S5. EPB41L4A-AS2 was downregulated in MDA-MB-231 breast cancer cells with ZNF217 overexpression in GEO dataset GSE35511. Figure S6. A Overlapping genes of EPB41L4A-AS2 correlated genes and paclitaxel related genes in BETMAN-TCM. B KEGG pathway analysis for EPB41L4A-AS2 correlated genes in BETMAN-TCM. C GO analysis for EPB41L4A-AS2 correlated genes in BETMAN-TCM.D OMIM analysis for EPB41L4A-AS2 correlated genes in BETMAN-TCM.E Pharmacological network analysis indicates that EPB41L4A-AS2 may be involved in paclitaxel related process in breast cancer. F Pharmacological network analysis indicates that EPB41L4A-AS2 may be involved in crosstalk with paclitaxel related genes in breast cancer. Figure S7. A Expression of EPB41L4A-AS2 in breast cancer cell lines. B&C overexpression efficiency of EPB41L4A-AS2 in UACC812 and BT549 cells. D Knockdown efficiency of EPB41L4A-AS2 in MDA-MB-453 cells. Figure S8. A-C Overexpression of each lncRNA (MEG3, WEE2-AS1 and HAND2-AS1) inhibited clone formation in UACC812 cells. D-F Overexpression of each lncRNA (MEG3, WEE2-AS1 and H [file 13046_2019_1096_MOESM2_ESM.zip › Figure S2.png]

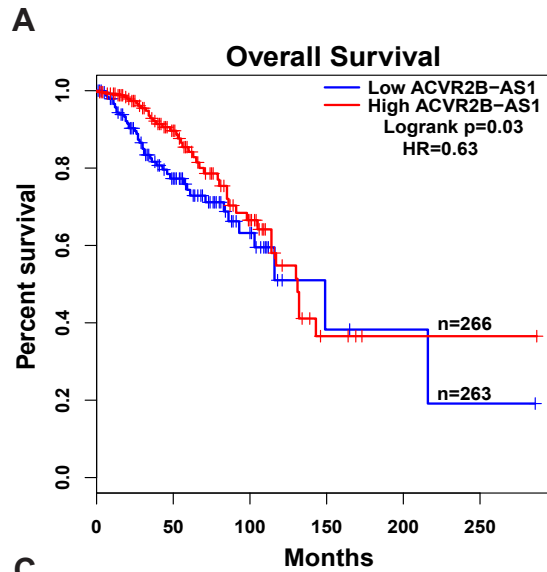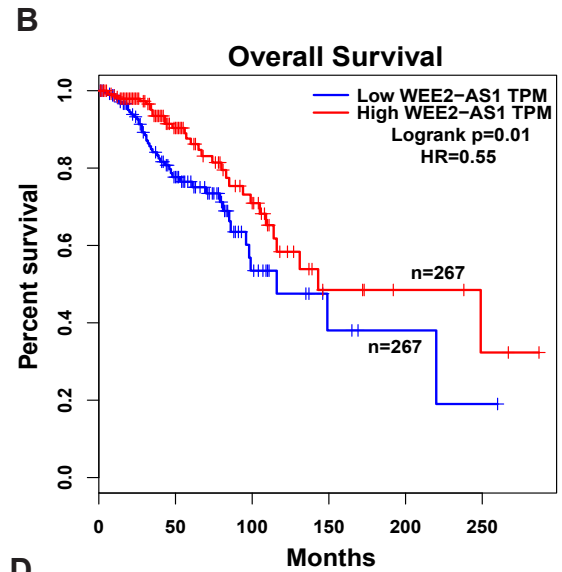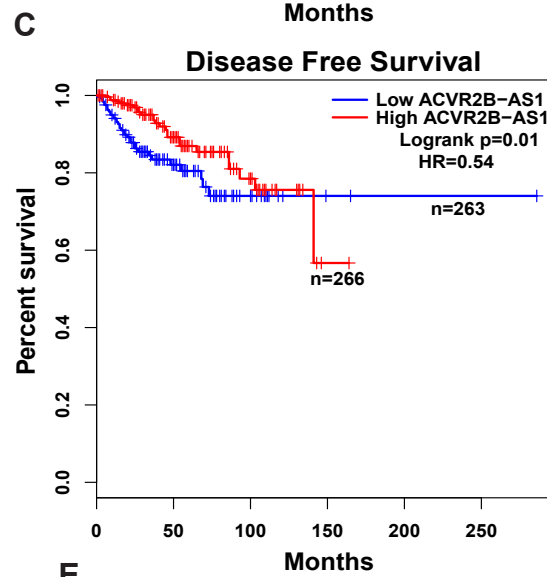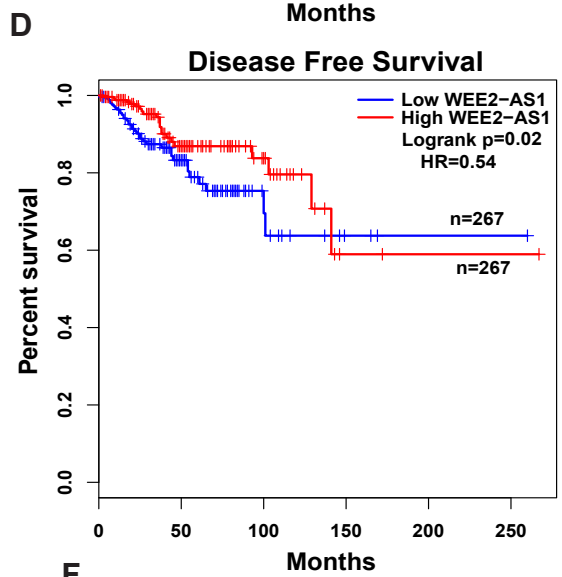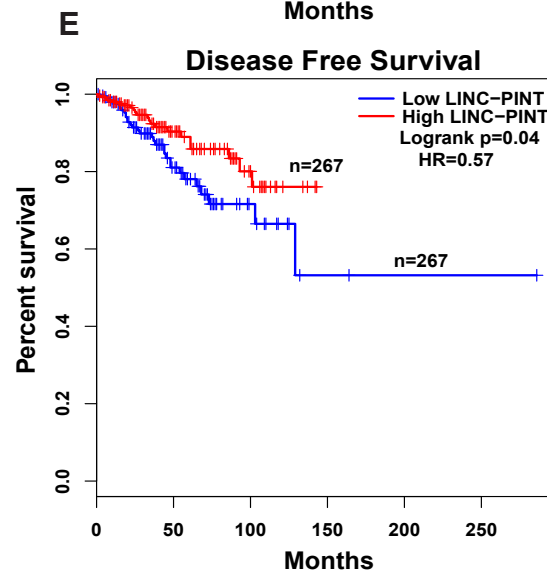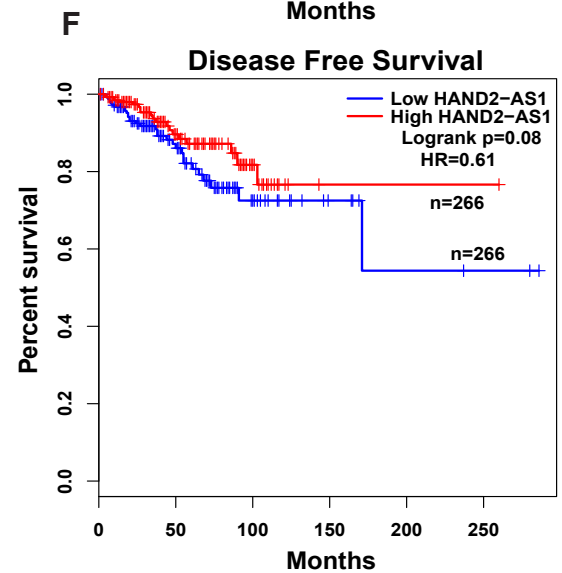

Supplement: Supplementary file 2 — Figure S1. TSLNR expression in breast cancer samples and normal tissue samples in HMUCC. Figure S2. Genetic alteration was also examined for these lncRNAs in breast cancer data in TCGA. Figure S3. A&B Patients with high expression (N = 266) of TSLNRs (ACVR2B-AS1 and WEE2-AS1) had favorable OS than those with low expression (N = 266) in breast cancer in TCGA. C-F Patients with high expression (N = 266) of TSLNRs (ACVR2B-AS1, WEE2-AS1, LINC-PINT and HAND2-AS1) had favorable DFS than those with low expression (N = 266) in breast cancer in TCGA. Figure S4. A-D Patients with high expression (N = 266) of TSLNRs (CYP1B1-AS1, LINC-PINT, LINC00667 and GRIK1-AS1) had favorable OS than those with low expression (N = 266) in breast cancer in TCGA. E-G Patients with high expression (N = 266) of TSLNRs (CYP1B1-AS1, FAM66C and GRIK1-AS1) had favorable DFS than those with low expression (N = 266) in breast cancer in TCGA. Figure S5. EPB41L4A-AS2 was downregulated in MDA-MB-231 breast cancer cells with ZNF217 overexpression in GEO dataset GSE35511. Figure S6. A Overlapping genes of EPB41L4A-AS2 correlated genes and paclitaxel related genes in BETMAN-TCM. B KEGG pathway analysis for EPB41L4A-AS2 correlated genes in BETMAN-TCM. C GO analysis for EPB41L4A-AS2 correlated genes in BETMAN-TCM.D OMIM analysis for EPB41L4A-AS2 correlated genes in BETMAN-TCM.E Pharmacological network analysis indicates that EPB41L4A-AS2 may be involved in paclitaxel related process in breast cancer. F Pharmacological network analysis indicates that EPB41L4A-AS2 may be involved in crosstalk with paclitaxel related genes in breast cancer. Figure S7. A Expression of EPB41L4A-AS2 in breast cancer cell lines. B&C overexpression efficiency of EPB41L4A-AS2 in UACC812 and BT549 cells. D Knockdown efficiency of EPB41L4A-AS2 in MDA-MB-453 cells. Figure S8. A-C Overexpression of each lncRNA (MEG3, WEE2-AS1 and HAND2-AS1) inhibited clone formation in UACC812 cells. D-F Overexpression of each lncRNA (MEG3, WEE2-AS1 and H [file 13046_2019_1096_MOESM2_ESM.zip › Figure S3.pdf]

**A**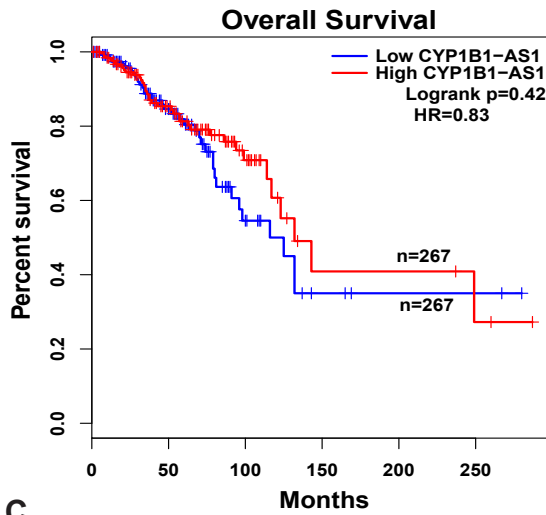**B**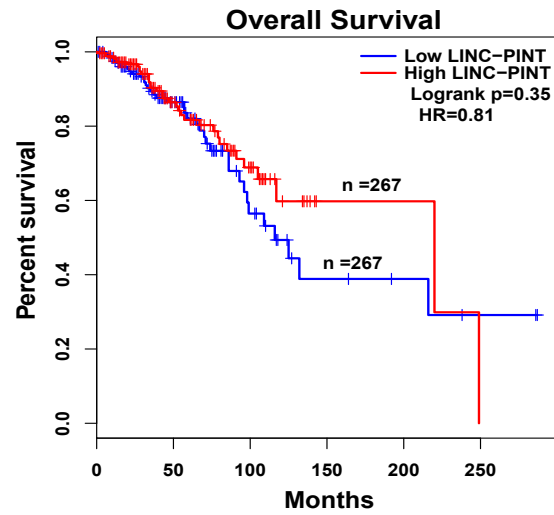**C**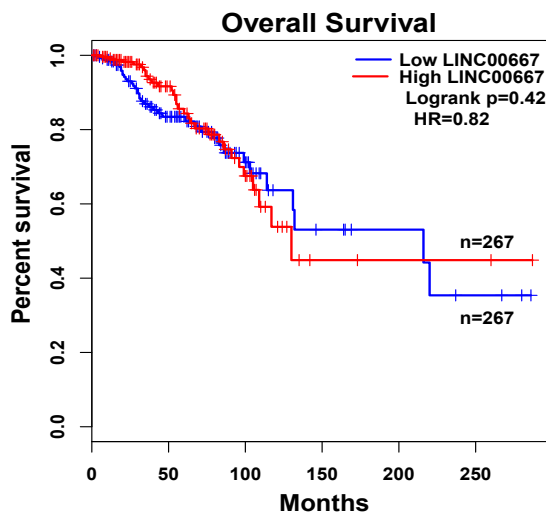**D**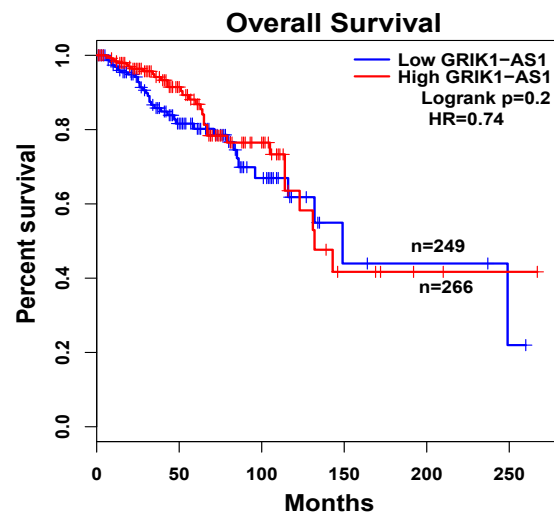**E**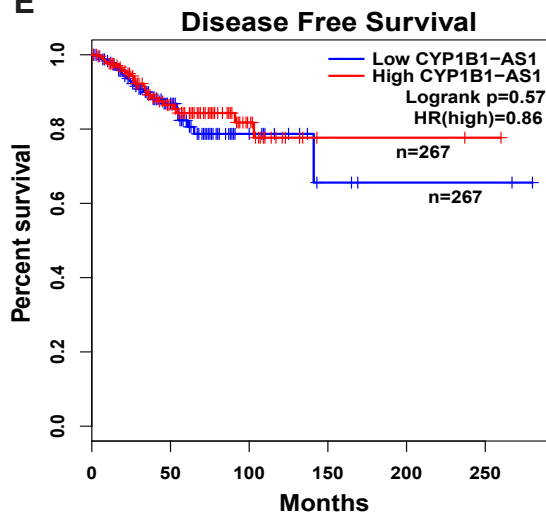**F**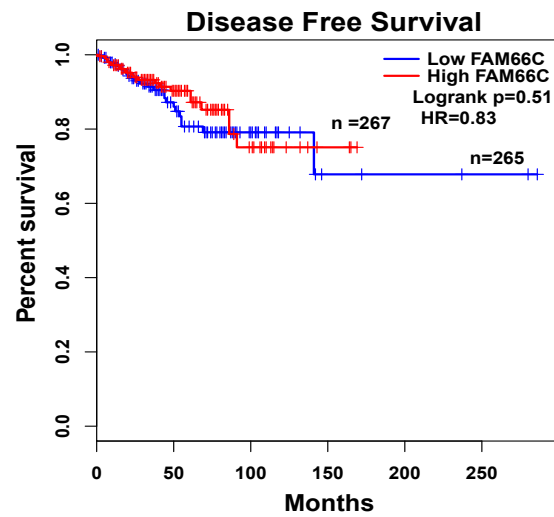**G**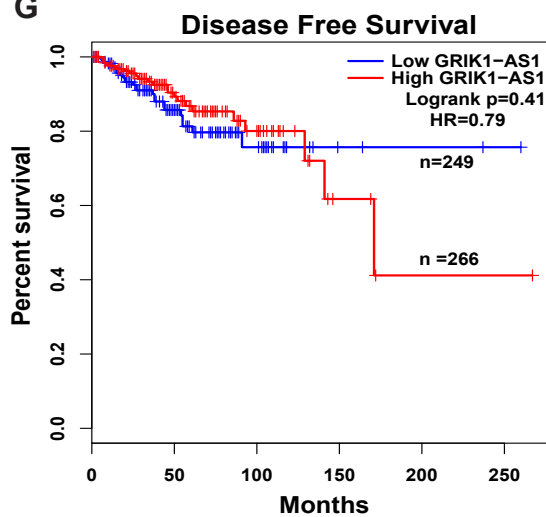

Supplement: Supplementary file 2 — Figure S1. TSLNR expression in breast cancer samples and normal tissue samples in HMUCC. Figure S2. Genetic alteration was also examined for these lncRNAs in breast cancer data in TCGA. Figure S3. A&B Patients with high expression (N = 266) of TSLNRs (ACVR2B-AS1 and WEE2-AS1) had favorable OS than those with low expression (N = 266) in breast cancer in TCGA. C-F Patients with high expression (N = 266) of TSLNRs (ACVR2B-AS1, WEE2-AS1, LINC-PINT and HAND2-AS1) had favorable DFS than those with low expression (N = 266) in breast cancer in TCGA. Figure S4. A-D Patients with high expression (N = 266) of TSLNRs (CYP1B1-AS1, LINC-PINT, LINC00667 and GRIK1-AS1) had favorable OS than those with low expression (N = 266) in breast cancer in TCGA. E-G Patients with high expression (N = 266) of TSLNRs (CYP1B1-AS1, FAM66C and GRIK1-AS1) had favorable DFS than those with low expression (N = 266) in breast cancer in TCGA. Figure S5. EPB41L4A-AS2 was downregulated in MDA-MB-231 breast cancer cells with ZNF217 overexpression in GEO dataset GSE35511. Figure S6. A Overlapping genes of EPB41L4A-AS2 correlated genes and paclitaxel related genes in BETMAN-TCM. B KEGG pathway analysis for EPB41L4A-AS2 correlated genes in BETMAN-TCM. C GO analysis for EPB41L4A-AS2 correlated genes in BETMAN-TCM.D OMIM analysis for EPB41L4A-AS2 correlated genes in BETMAN-TCM.E Pharmacological network analysis indicates that EPB41L4A-AS2 may be involved in paclitaxel related process in breast cancer. F Pharmacological network analysis indicates that EPB41L4A-AS2 may be involved in crosstalk with paclitaxel related genes in breast cancer. Figure S7. A Expression of EPB41L4A-AS2 in breast cancer cell lines. B&C overexpression efficiency of EPB41L4A-AS2 in UACC812 and BT549 cells. D Knockdown efficiency of EPB41L4A-AS2 in MDA-MB-453 cells. Figure S8. A-C Overexpression of each lncRNA (MEG3, WEE2-AS1 and HAND2-AS1) inhibited clone formation in UACC812 cells. D-F Overexpression of each lncRNA (MEG3, WEE2-AS1 and H [file 13046_2019_1096_MOESM2_ESM.zip › Figure S4.pdf]

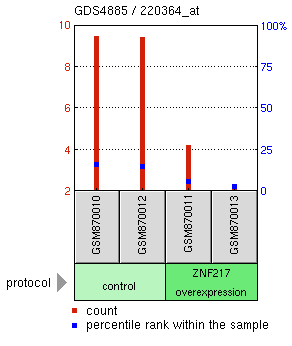

Supplement: Supplementary file 2 — Figure S1. TSLNR expression in breast cancer samples and normal tissue samples in HMUCC. Figure S2. Genetic alteration was also examined for these lncRNAs in breast cancer data in TCGA. Figure S3. A&B Patients with high expression (N = 266) of TSLNRs (ACVR2B-AS1 and WEE2-AS1) had favorable OS than those with low expression (N = 266) in breast cancer in TCGA. C-F Patients with high expression (N = 266) of TSLNRs (ACVR2B-AS1, WEE2-AS1, LINC-PINT and HAND2-AS1) had favorable DFS than those with low expression (N = 266) in breast cancer in TCGA. Figure S4. A-D Patients with high expression (N = 266) of TSLNRs (CYP1B1-AS1, LINC-PINT, LINC00667 and GRIK1-AS1) had favorable OS than those with low expression (N = 266) in breast cancer in TCGA. E-G Patients with high expression (N = 266) of TSLNRs (CYP1B1-AS1, FAM66C and GRIK1-AS1) had favorable DFS than those with low expression (N = 266) in breast cancer in TCGA. Figure S5. EPB41L4A-AS2 was downregulated in MDA-MB-231 breast cancer cells with ZNF217 overexpression in GEO dataset GSE35511. Figure S6. A Overlapping genes of EPB41L4A-AS2 correlated genes and paclitaxel related genes in BETMAN-TCM. B KEGG pathway analysis for EPB41L4A-AS2 correlated genes in BETMAN-TCM. C GO analysis for EPB41L4A-AS2 correlated genes in BETMAN-TCM.D OMIM analysis for EPB41L4A-AS2 correlated genes in BETMAN-TCM.E Pharmacological network analysis indicates that EPB41L4A-AS2 may be involved in paclitaxel related process in breast cancer. F Pharmacological network analysis indicates that EPB41L4A-AS2 may be involved in crosstalk with paclitaxel related genes in breast cancer. Figure S7. A Expression of EPB41L4A-AS2 in breast cancer cell lines. B&C overexpression efficiency of EPB41L4A-AS2 in UACC812 and BT549 cells. D Knockdown efficiency of EPB41L4A-AS2 in MDA-MB-453 cells. Figure S8. A-C Overexpression of each lncRNA (MEG3, WEE2-AS1 and HAND2-AS1) inhibited clone formation in UACC812 cells. D-F Overexpression of each lncRNA (MEG3, WEE2-AS1 and H [file 13046_2019_1096_MOESM2_ESM.zip › Figure S5.tif]

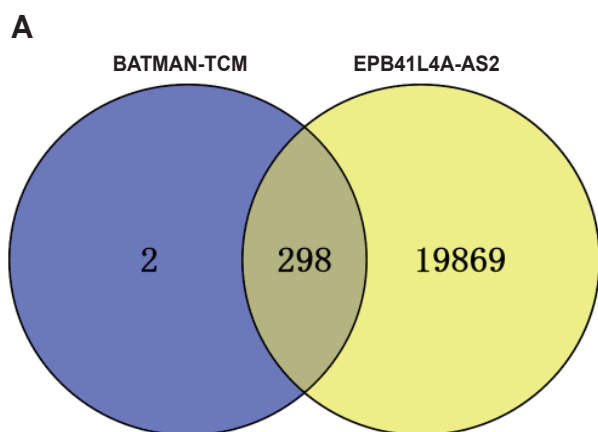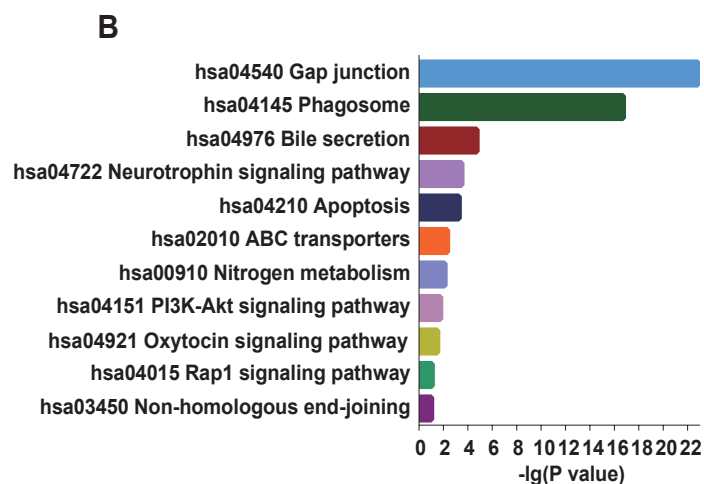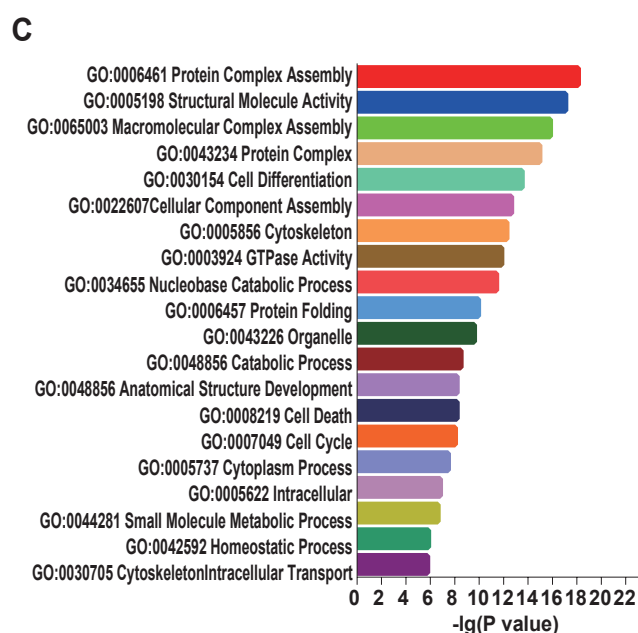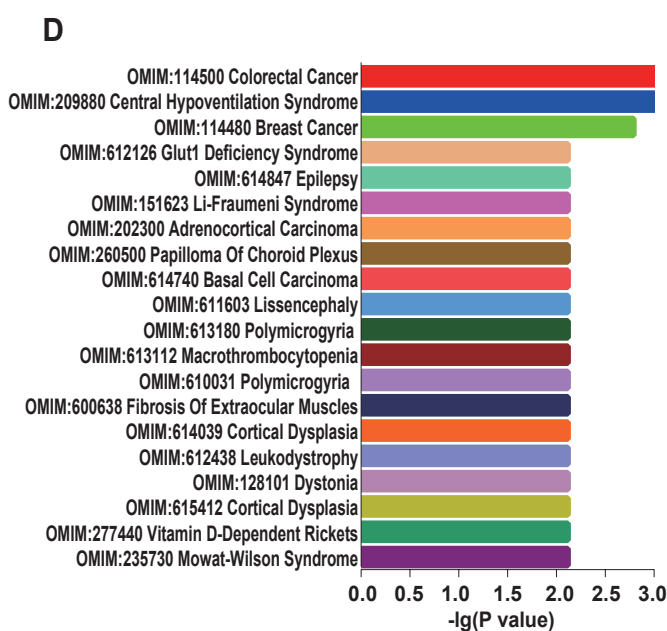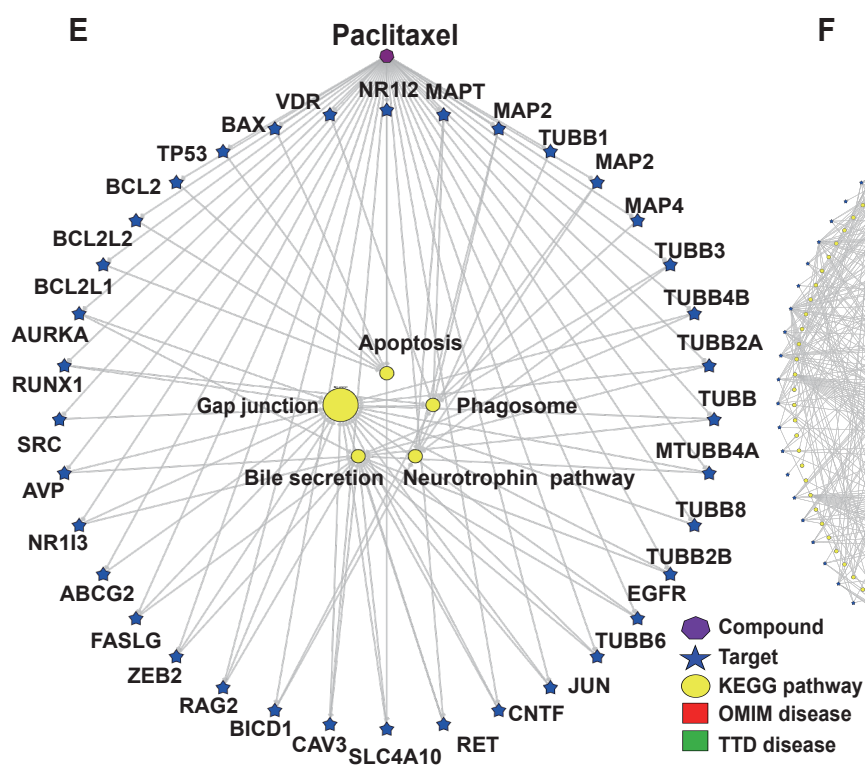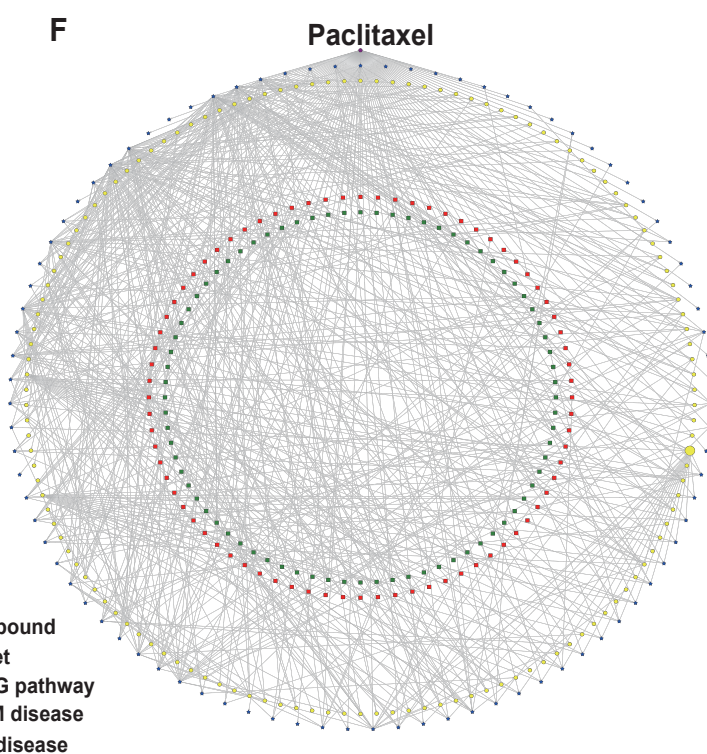

Supplement: Supplementary file 2 — Figure S1. TSLNR expression in breast cancer samples and normal tissue samples in HMUCC. Figure S2. Genetic alteration was also examined for these lncRNAs in breast cancer data in TCGA. Figure S3. A&B Patients with high expression (N = 266) of TSLNRs (ACVR2B-AS1 and WEE2-AS1) had favorable OS than those with low expression (N = 266) in breast cancer in TCGA. C-F Patients with high expression (N = 266) of TSLNRs (ACVR2B-AS1, WEE2-AS1, LINC-PINT and HAND2-AS1) had favorable DFS than those with low expression (N = 266) in breast cancer in TCGA. Figure S4. A-D Patients with high expression (N = 266) of TSLNRs (CYP1B1-AS1, LINC-PINT, LINC00667 and GRIK1-AS1) had favorable OS than those with low expression (N = 266) in breast cancer in TCGA. E-G Patients with high expression (N = 266) of TSLNRs (CYP1B1-AS1, FAM66C and GRIK1-AS1) had favorable DFS than those with low expression (N = 266) in breast cancer in TCGA. Figure S5. EPB41L4A-AS2 was downregulated in MDA-MB-231 breast cancer cells with ZNF217 overexpression in GEO dataset GSE35511. Figure S6. A Overlapping genes of EPB41L4A-AS2 correlated genes and paclitaxel related genes in BETMAN-TCM. B KEGG pathway analysis for EPB41L4A-AS2 correlated genes in BETMAN-TCM. C GO analysis for EPB41L4A-AS2 correlated genes in BETMAN-TCM.D OMIM analysis for EPB41L4A-AS2 correlated genes in BETMAN-TCM.E Pharmacological network analysis indicates that EPB41L4A-AS2 may be involved in paclitaxel related process in breast cancer. F Pharmacological network analysis indicates that EPB41L4A-AS2 may be involved in crosstalk with paclitaxel related genes in breast cancer. Figure S7. A Expression of EPB41L4A-AS2 in breast cancer cell lines. B&C overexpression efficiency of EPB41L4A-AS2 in UACC812 and BT549 cells. D Knockdown efficiency of EPB41L4A-AS2 in MDA-MB-453 cells. Figure S8. A-C Overexpression of each lncRNA (MEG3, WEE2-AS1 and HAND2-AS1) inhibited clone formation in UACC812 cells. D-F Overexpression of each lncRNA (MEG3, WEE2-AS1 and H [file 13046_2019_1096_MOESM2_ESM.zip › Figure S6.pdf]

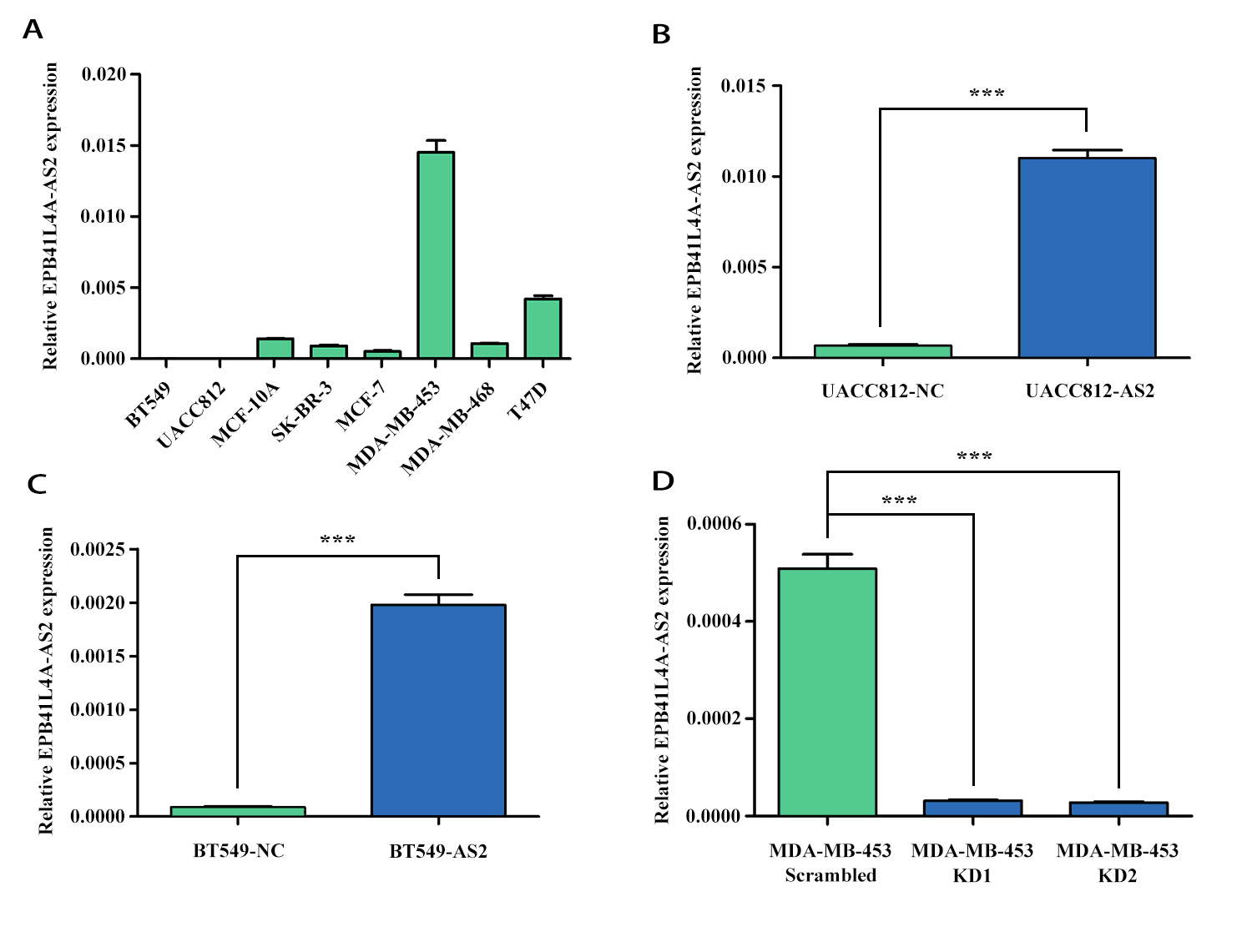

Supplement: Supplementary file 2 — Figure S1. TSLNR expression in breast cancer samples and normal tissue samples in HMUCC. Figure S2. Genetic alteration was also examined for these lncRNAs in breast cancer data in TCGA. Figure S3. A&B Patients with high expression (N = 266) of TSLNRs (ACVR2B-AS1 and WEE2-AS1) had favorable OS than those with low expression (N = 266) in breast cancer in TCGA. C-F Patients with high expression (N = 266) of TSLNRs (ACVR2B-AS1, WEE2-AS1, LINC-PINT and HAND2-AS1) had favorable DFS than those with low expression (N = 266) in breast cancer in TCGA. Figure S4. A-D Patients with high expression (N = 266) of TSLNRs (CYP1B1-AS1, LINC-PINT, LINC00667 and GRIK1-AS1) had favorable OS than those with low expression (N = 266) in breast cancer in TCGA. E-G Patients with high expression (N = 266) of TSLNRs (CYP1B1-AS1, FAM66C and GRIK1-AS1) had favorable DFS than those with low expression (N = 266) in breast cancer in TCGA. Figure S5. EPB41L4A-AS2 was downregulated in MDA-MB-231 breast cancer cells with ZNF217 overexpression in GEO dataset GSE35511. Figure S6. A Overlapping genes of EPB41L4A-AS2 correlated genes and paclitaxel related genes in BETMAN-TCM. B KEGG pathway analysis for EPB41L4A-AS2 correlated genes in BETMAN-TCM. C GO analysis for EPB41L4A-AS2 correlated genes in BETMAN-TCM.D OMIM analysis for EPB41L4A-AS2 correlated genes in BETMAN-TCM.E Pharmacological network analysis indicates that EPB41L4A-AS2 may be involved in paclitaxel related process in breast cancer. F Pharmacological network analysis indicates that EPB41L4A-AS2 may be involved in crosstalk with paclitaxel related genes in breast cancer. Figure S7. A Expression of EPB41L4A-AS2 in breast cancer cell lines. B&C overexpression efficiency of EPB41L4A-AS2 in UACC812 and BT549 cells. D Knockdown efficiency of EPB41L4A-AS2 in MDA-MB-453 cells. Figure S8. A-C Overexpression of each lncRNA (MEG3, WEE2-AS1 and HAND2-AS1) inhibited clone formation in UACC812 cells. D-F Overexpression of each lncRNA (MEG3, WEE2-AS1 and H [file 13046_2019_1096_MOESM2_ESM.zip › Figure S7.tif]

**A**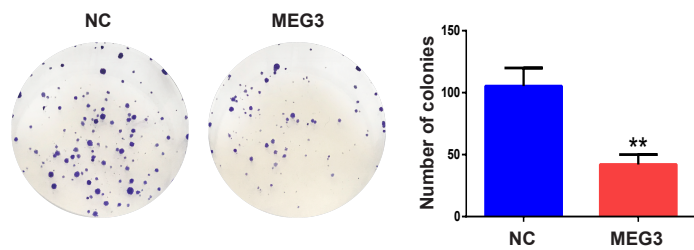**B**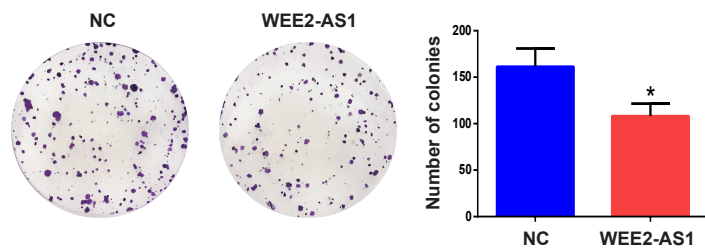**C**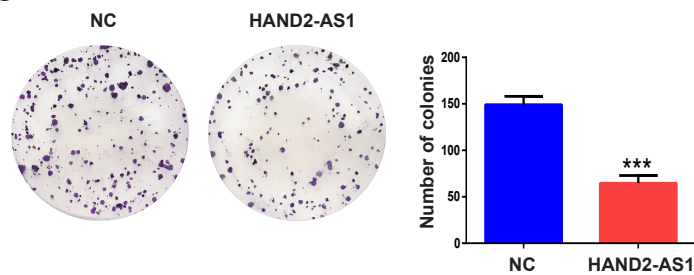**D**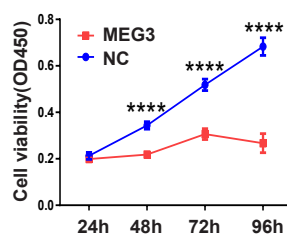**E**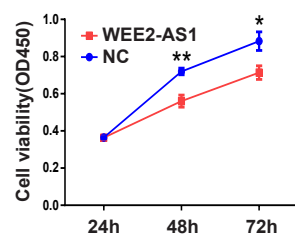**F**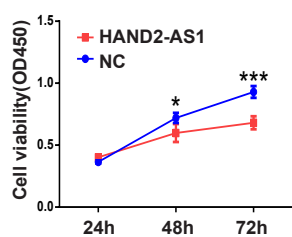

Supplement: Supplementary file 2 — Figure S1. TSLNR expression in breast cancer samples and normal tissue samples in HMUCC. Figure S2. Genetic alteration was also examined for these lncRNAs in breast cancer data in TCGA. Figure S3. A&B Patients with high expression (N = 266) of TSLNRs (ACVR2B-AS1 and WEE2-AS1) had favorable OS than those with low expression (N = 266) in breast cancer in TCGA. C-F Patients with high expression (N = 266) of TSLNRs (ACVR2B-AS1, WEE2-AS1, LINC-PINT and HAND2-AS1) had favorable DFS than those with low expression (N = 266) in breast cancer in TCGA. Figure S4. A-D Patients with high expression (N = 266) of TSLNRs (CYP1B1-AS1, LINC-PINT, LINC00667 and GRIK1-AS1) had favorable OS than those with low expression (N = 266) in breast cancer in TCGA. E-G Patients with high expression (N = 266) of TSLNRs (CYP1B1-AS1, FAM66C and GRIK1-AS1) had favorable DFS than those with low expression (N = 266) in breast cancer in TCGA. Figure S5. EPB41L4A-AS2 was downregulated in MDA-MB-231 breast cancer cells with ZNF217 overexpression in GEO dataset GSE35511. Figure S6. A Overlapping genes of EPB41L4A-AS2 correlated genes and paclitaxel related genes in BETMAN-TCM. B KEGG pathway analysis for EPB41L4A-AS2 correlated genes in BETMAN-TCM. C GO analysis for EPB41L4A-AS2 correlated genes in BETMAN-TCM.D OMIM analysis for EPB41L4A-AS2 correlated genes in BETMAN-TCM.E Pharmacological network analysis indicates that EPB41L4A-AS2 may be involved in paclitaxel related process in breast cancer. F Pharmacological network analysis indicates that EPB41L4A-AS2 may be involved in crosstalk with paclitaxel related genes in breast cancer. Figure S7. A Expression of EPB41L4A-AS2 in breast cancer cell lines. B&C overexpression efficiency of EPB41L4A-AS2 in UACC812 and BT549 cells. D Knockdown efficiency of EPB41L4A-AS2 in MDA-MB-453 cells. Figure S8. A-C Overexpression of each lncRNA (MEG3, WEE2-AS1 and HAND2-AS1) inhibited clone formation in UACC812 cells. D-F Overexpression of each lncRNA (MEG3, WEE2-AS1 and H [file 13046_2019_1096_MOESM2_ESM.zip › Figure S8.pdf]
